# High-throughput sequencing detected a virus–viroid complex in a single pokeweed plant

**DOI:** 10.3389/fpls.2024.1435611

**Published:** 2024-08-22

**Authors:** Myeonghwan Kwak, Elisa Troiano, Eui-Joon Kil, Giuseppe Parrella

**Affiliations:** ^1^ Department of Plant Medicals, Andong National University, Andong, Republic of Korea; ^2^ Institute for Sustainable Plant Protection, National Research Council, Portici, Italy

**Keywords:** next generation sequencing, *Polerovirus*, TuYV, CEVd, *Phytolacca americana*, emerging viruses

## Abstract

In this study, total RNA high-throughput sequencing (HTS) of a single symptomatic *Phytolacca americana* plant enabled the obtention of a nearly complete genome of two new isolates of turnip yellows virus (TuYV), named TuYV-ITA1 and TuYV-ITA2, and revealed a mixed infection with a new variant of citrus exocortis viroid (CEVd), named CEVd-ITA1. The TuYV-ITA2 isolate diverged from the known virus isolates of TuYV and showed variability in the P0 and P5 readthrough domain. Recombination analysis revealed its recombinant nature between TuYV and an unidentified polerovirus. The putative recombination event was identified in the P5 readthrough domain of the TuYMV-ITA2 isolate. Our results thus represent the first report of TuYV in Italy and some molecular evidence for the possible natural co-existence of TuYV and CEVd in a new natural host for both infectious entities. This study is adding further knowledge about the role of weed plants as virus reservoirs, and thus additional biological and impact studies would be desirable to determine in particular the role of *P. americana* in the spread of TuYV and if this virus should be considered a new threat for the susceptible Italian crops.

## 1 Introduction

*Phytolacca americana* (family Phytolaccaceae), also known as pokeweed, American pokeweed, dragonberries, or Turkish berry, is a perennial plant native to the eastern part of North America, Asia, and New Zealand. It was imported during the 17th century in Europe for its ornamental value and became naturalized. Pokeweed can grow up to 2.5 m and has vigorous stems, more or less colored red, and has elliptic leaves of over 20 cm in length, which take on a reddish color in autumn. It has white or pinkish flowers forming large spikes, followed by berries that are first green and then turn blackish purple. Pokeweed has a long history for use as a food, homeopathic medicine, herb, clothing dye, writing ink, wine colorant, and much more. Although it is also used for food purposes, extreme caution must be taken, as the plant is poisonous in all its parts and can cause severe symptoms, including death in rare cases. Pokeweed is sometimes grown as an ornamental or garden vegetable; however, it is more often considered an undesirable weed and pest species by farmers (Hausner and Poppenga, 2013; Oneto, 2018).

The pokeweed antiviral protein (PAP), isolated from the leaves of pokeweed, displays a broad spectrum of antiviral activity against plant and animal viruses, thanks to its ability to depurinate and thus damage ribosomes (Irvin and Uckun, 1992; Katalin et al., 1999). Since virus replication depends on the host machinery, ribosome-damaged host cells will not be able to support virus replication (Lodge et al., 1993). Nevertheless, *P. americana* is susceptible to some viruses that have been detected and isolated from this plant species, including cucumber mosaic virus (CMV), pokeweed mosaic virus (PoMV), tobacco mosaic virus (TMV), dodder latent virus (DLV), and, recently, parietaria mottle virus (PMoV) (Xia et al., 1988; Sastry et al., 2019; Parrella et al., 2021). In October 2021, virus-like symptoms were observed in a pokeweed plant identified in a public park located in the province of Naples (Southern Italy). Preliminary investigation for virus search by generic primers revealed turnip yellows virus (TuYV, genus *Polerovirus*, family *Solemoviridae*) infection associated to the symptomatic pokeweed (Sõmera et al., 2021). In this study, using high-throughput sequencing (HTS), we confirmed the infection by two divergent isolates of TuYV and also revealed a mixed infection with a new citrus exocortis viroid (CEVd) isolate. We also employed RT-PCR to confirm the HTS results and the coexistence of TuYV and CEVd new variants. Furthermore, we studied the phylogenetic relationships among these newly described isolates and other globally reported isolates of TuYV and its closely related brassica yellows virus (BrYV) isolates, now considered as the Asian variant of the TuYV based on the close molecular relationship between TuYV and BrYV isolates described so far (Peng et al., 2023).

TuYV has a wide host range and, experimentally, can infect species from at least 23 plant families, including many species of agronomic importance (Slavíková et al., 2022). The virus is of particular interest as a pathogen of oilseed rape (Smith and Hinckes, 1985; Hill et al., 1989; Jay et al., 1999) but is also of economic importance in lettuce (Walkey and Payne, 1990; Walkey and Pink, 1990). The diverse range of cultivated plants and weed species susceptible to TuYV increases the potential reservoir of hosts in which the virus can survive throughout the winter and provides a source for future virus outbreaks (Smith and Hinckes, 1985; Stevens et al., 1994; Latham et al., 2003). The results of our study provide valuable further information about the genomic diversity and recombination dynamics of TuYV, highlighting the role of pokeweed as a reservoir of genetic variability in nature of this potentially destructive virus as described for oilseed rape (Peng et al., 2023).

## 2 Materials and methods

### 2.1 Plant source material and nucleic acid extraction

**Figure 1:**
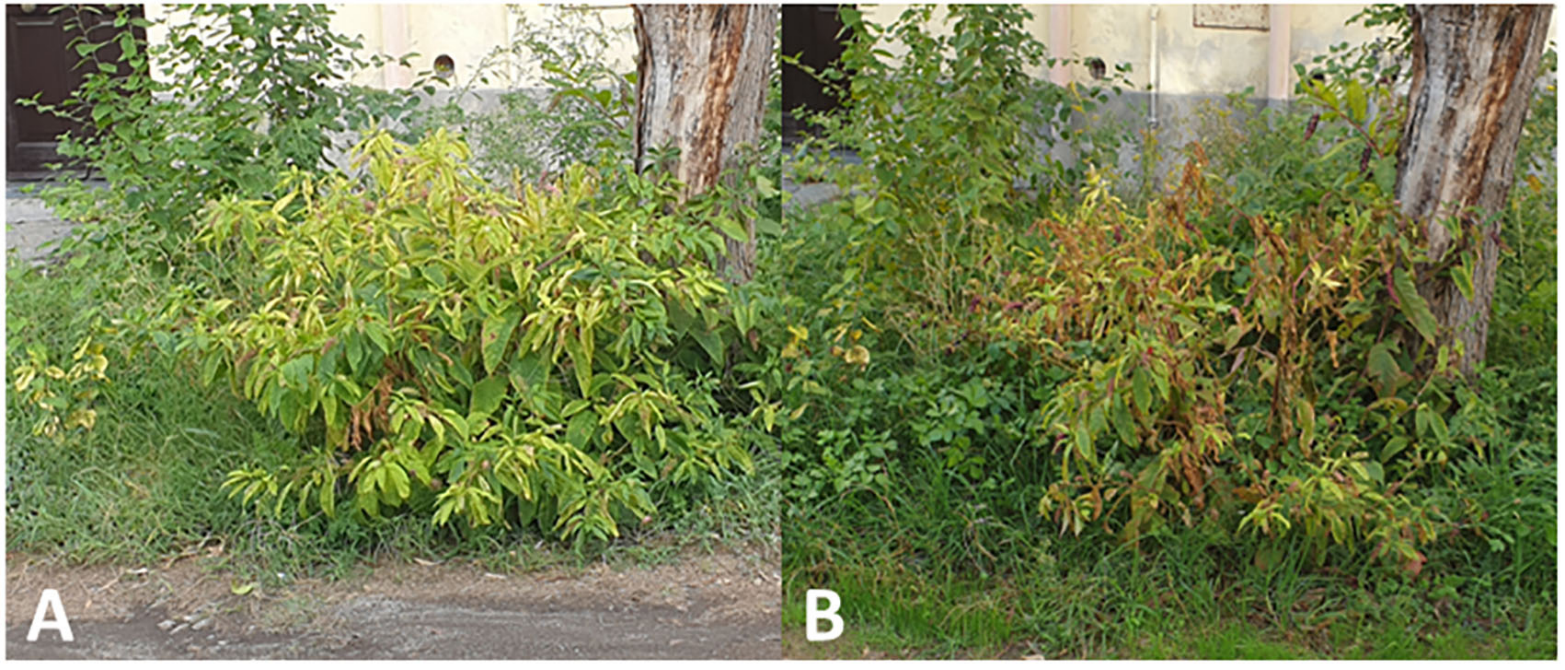
Evolution and symptom development on *Phytolacca americana* plant found infected with turnip yellows virus (TuYV) and citrus exocortis viroid (CEVd): yellowing **(A)** followed by dieback and wilting **(B)**.

An approximately 2-year-old pokeweed growing spontaneously in the Giovanni Gussone Park, located in Portici municipality (coordinates: 40°48′55″ N, 14°21′04″ E) of the metropolitan city of Naples (Campania region, Southern Italy), and showing pronounced chlorosis at the beginning of October 2021 (**Figure 1A**) was selected for HTS analysis. This plant showed dieback and wilting 1 month later (**Figure 1B**) and died within a few days. Extraction of total RNA was carried out with TRI-reagent (Sigma-Aldrich, USA) using 100 mg of fresh leaves in order to obtain a sufficient amount of RNA to submit to RT-PCR and HTS analysis.

### 2.2 Preliminary molecular screening for virus infection

The presence of TSWV, INSV, CMV, PMoV, poleroviruses, and potyviruses in the collected sample was explored through virus-specific RT-PCR assays. cDNA was synthesized from the extracted RNAs using the ImProm-II reverse transcriptase system (Promega, USA) according to the manufacturer’s instructions. Reactions were performed at 42°C for 60 min, followed by incubation at 70°C for 5 min. The PCR conditions and cycles are described in Mumford et al. (1996), Adkins and Rosskopf (2002); Knierim et al. (2014); Parrella et al. (2021), and Gibbs and Mackenzie (1997).

### 2.3 High-throughput sequencing and sequence analysis

Ribosomal RNA was depleted from the purified RNA using the RiboMinus Plant Kit for RNA-Seq (Thermo Fisher Scientific, Waltham, MA, USA), and sequencing libraries were prepared by using TruSeq stranded total RNA with the RiboZero Plant kit (Illumina, San Diego, CA, USA), while high-throughput sequencing was performed on the Illumina NovaSeq 6000 platform, with 150 nt paired-end chemistry by Macrogen company (Republic of Korea). The bioinformatics analysis of the obtained raw data was performed using the CLC Genomics Workbench (Qiagen, Hilden, Germany). The reads obtained were trimmed and filtrated (reads with *Q* ≤ 25 and shorter than 15 bp were discarded). The trimmed sequencing reads were first mapped to NCBI viral RefSeq database (November 2023). Additionally, *de novo* assembly was performed to detect novel viruses and mutant viruses, and the assembled contigs were annotated by BLASTn search to NCBI GenBank. The reads were then mapped to the corresponding most similar viral genome sequences to the contigs from NCBI GenBank (**Supplementary Table S1**).

### 2.4 Validation of HTS results by RT-PCR detection of pokeweed-infecting TuYV and CEVd

A specific couple of primers was also designed to confirm the presence of two different TuYV isolates within the infected pokeweed (**Supplementary Table S2**). cDNAs were produced as described above (see Section 2.2), while PCR amplifications were performed using the proofreading Platinum SuperFi II DNA Polymerase (Thermo Fisher Scientific Inc., Waltham, MA, USA) under the following cycling conditions: initial denaturation at 94°C for 4 min; 35 cycles of 94°C for 30 s, 56°C for 30 s, and 72°C for 1 min; and final extension step at 72°C for 10 min. All PCR products were directly sequenced in both senses (Microsynth, Seqlab GmbH, Göttingen, Germany). Scaffolds were finally assembled by mapping the TuYV and CEVd contigs and residual reads on the TuYV and CEVd reference genomes. Finally, the consensus viral genomes were deposited in NCBI GenBank and used for further analyses.

### 2.5 Phylogenetic relationship and variability of TuYV isolates

The TuY-ITA1 and TuY-ITA2 sequences were firstly aligned using the on-line tool EMBOSS Needle (https://www.ebi.ac.uk/jdispatcher/psa/emboss_needle), which creates an optimal global alignment of two sequences using the Needleman-Wunsch algorithm and MAFFT (version 7; https://mafft.cbrc.jp/alignment/server), which provide a dot plot between two sequences (not shown). Multiple alignments with the distance matrix for both nucleotide and amino and phylogenetic tree reconstruction were performed using ClustalW implemented in MEGA X (Kumar et al., 2018) on the obtained TuYV sequences and on the sequences of TuYV and the closely related brassica yellows virus (BrYV) available in the GenBank database (www.ncbi.nlm.nih.gov) listed in **Supplementary Table S1**. In particular, phylogeny was inferred from the whole-genome nucleotide sequence of TuYV and BrYV using the maximum likelihood method implemented in MEGA X and from P0 and P1–P2 proteins and, because of their variability (Filardo et al., 2021), from the P3 (coat protein) and P3–P5 (readthrough domain) proteins of poleroviruses listed in **Supplementary Table S2**. The mean pairwise number of nucleotide differences per site (nucleotide diversity, π) was estimated with DnaSPv.6.10 (Rozas et al., 2017), using a 100-nt sliding window with a step size of 10 nt across the sequences of the two TuYV isolates each aligned with their respective top four viruses according to BLASTn results.

### 2.6 Recombination analysis

The alignments of whole-genome sequences of reference poleroviruses species, including TuYV-ITA1 and TuYV-ITA2, were examined for the presence of intra- and inter-species recombination using Recombination Detection Program, version 4.101 (RDP4). The recombination analysis was performed using all seven detection methods implemented in RDP4 (RDP, GENECONV, Chimera, MaxChi, BootScan, SiScan, and 3Seq) using default parameters. Potential recombination events were considered statistically significant when supported by at least four different methods with a *p*-value lower than a Bonferroni-corrected cutoff of 0.05. In addition, a BLASTn search was performed on the recombinant region to check this genomic region with all the poleroviruses variability available in GeneBank, in order to find putative parentals of the recombinant sequence, since both intraspecific and interspecific RNA recombination events are frequent in poleroviruses (Pagan and Holmes, 2010).

### 2.7 Molecular characterization of the CEVd isolate

The CEVd-ITA1 sequence was submitted to BLAST search to identify homologous sequences in NCBI databases (http://www.ncbi.nlm.nih.gov/) (McGinnis and Madden, 2004). CEVd-ITA1 was then aligned with those of CEVd class A and class B reference strains using Multalin. The secondary structure was predicted with the aid of the *mfold* software (http://mfold2.wustl.edu/mfold/rna/form; circular version) and compared with those described previously (Bernad et al., 2005).

## 3 Results

### 3.1 HTS data analyses and identification of TuYV and CEVd

**Figure 2:**
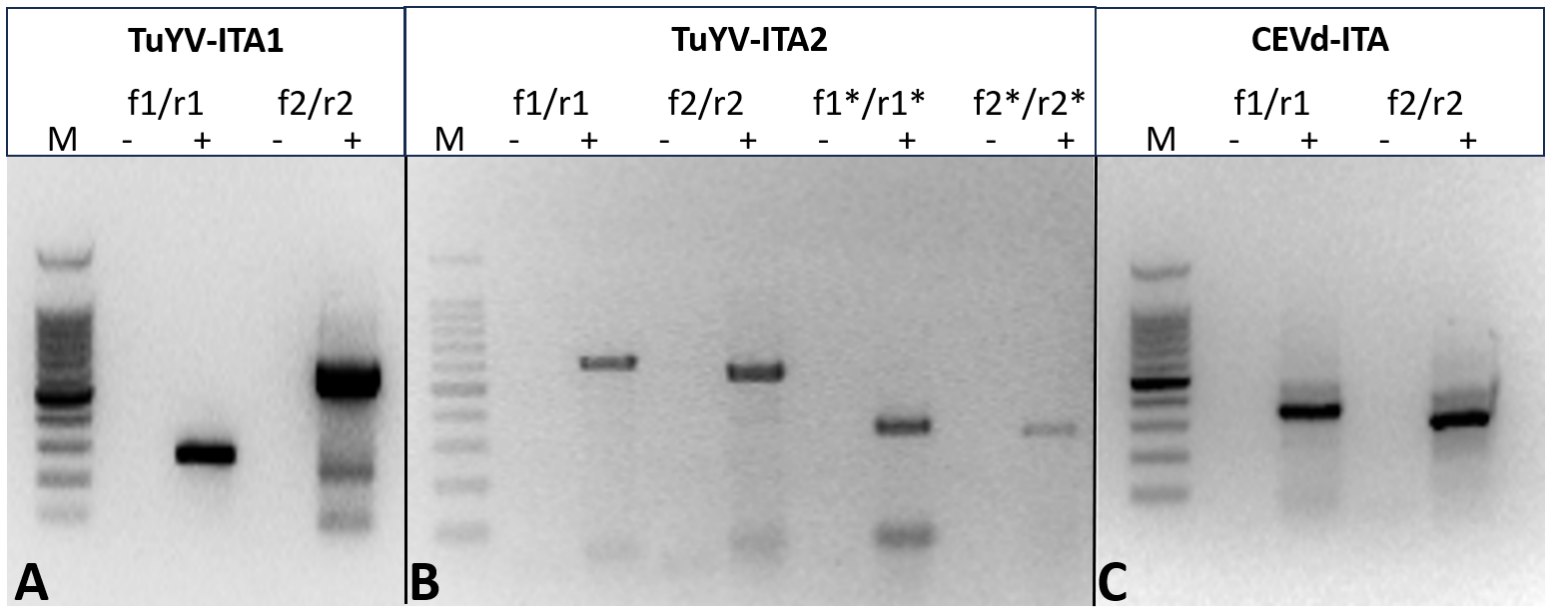
Different tails of the RT-PCR fragment obtained with primer pairs designed to amplify specifically TuYV-ITA1 **(A)** and TuYV-ITA2 **(B)** based on the nucleotide differences identified by HTS in the sequences of the two isolates. The expected tails of amplicons were 282 bp for TuYV1-f1/TuYV-r1 and 629 bp for TuYV1-f2/TuYV1-r2 (isolate TuYV-ITA1; **Figure 1A**) as well as 604 bp for TuYV2-f1/TuYV2-r1, 540 bp for TuYV2-f2/TuYV2-r2, 327 bp for TuYV2-f1*/TuYV2-r1*, and 310 bp for TuYV2-f2*/TuYV2-r2* (isolate TuYV-ITA2; **Figure 1B**). The alignment between TuYV-ITA1 and TuYV-ITA2 with the sequences and positions of the primers used is shown in **Supplementary Figure S2**. **(C)** Amplicons obtained by RT-PCR with two sets of primer pairs designed on the CEVD-ITA1 sequence based on the HTS results. The expected tails of amplicons were 350 bp for CEVd-f1/CEVd-r1 and 301 for CEVd-f2/CEVd-r2. M, 100-bp DNA ladder; -, negative control; +, RNA from infected pokeweed. The primer sequences and the expected tails are listed in **Supplementary Table S4**. (This photo was generated by assembling three different gels whose original photos are reported as “Note to **Figure 2**” in the **Supplementary Material**).

In total, 28,848,885 reads were obtained from the symptomatic *P. americana* plant, reduced to 27,162,632 after trimming for quality. Of these, 374,986 were related to viruses and 111,757 to viroids. All the virus-related sequence reads were mapped to turnip yellows virus (TuYV) and citrus exocortis viroid (CEVd). Two isolates of TuYV (named TuYV-ITA1 and TuYV-ITA2) and one isolate of CEVd (CEVd_ITA1) were definitely assembled, and the sequences were subsequently deposited in the GenBank database with accession numbers OQ632303, OQ632304, and OQ632305, respectively. Among the two TuYV isolates, TuYV-ITA1 consisted of 5,631 nt and TuYV-ITA2 consisted of 5,655 nt. A quantitative analysis of viral read was performed by mapping the trimmed reads with over 99% similarity to each assembled viral genome. The most abundant was TuYV-ITA1 with 866,392 reads, followed by TuYB-ITA2 with 354,217 reads, and CEVd was the least abundant with 75,061 reads (**Supplementary Figure S1**). The BLASTn analysis revealed that TuYV-ITA1 had the highest percentage of nucleotide identity with the TuYV isolate TuYV-FL1 (acc. no. NC_003743), while TuYV-ITA2 had the highest identity (92.1% of identity with 91% of genomic cover) not only with the HN/tobacco/2017 isolate of TuYV (MK616236) but also with the two isolates of BrYV, Anhui (MF314820, with 92.3% of identity with 89% of genomic cover) and BrYV-NtabQJ (MK057527, with 92.2% of identity and 91% of genomic cover). The different tails of the RT-PCR amplicons, obtained by using specific primers designed on the divergent genomic regions between the two isolates, confirmed the genomic differences among them (**Figures 2A, B**; **Supplementary Table S2**; **Supplementary Figure S2**). The amplicons of the expected tails, obtained by RT-PCR with a couple of specific primers for CEVd, confirmed the virus–viroid co-infection in the pokeweed plant (**Figure 2C**). All the amplicons obtained by RT-PCR were Sanger-sequenced, and the sequences were 100% identical to those obtained with HTS and relative to TuYV-ITA1, TuYV-ITA2, and CEVd-ITA1, respectively.

### 3.2 Phylogenetic affinities of pokeweed TuYV isolates, sequence analysis, and evidence for the recombinant nature of the TuYV-ITA2 isolate

**Figure 3:**
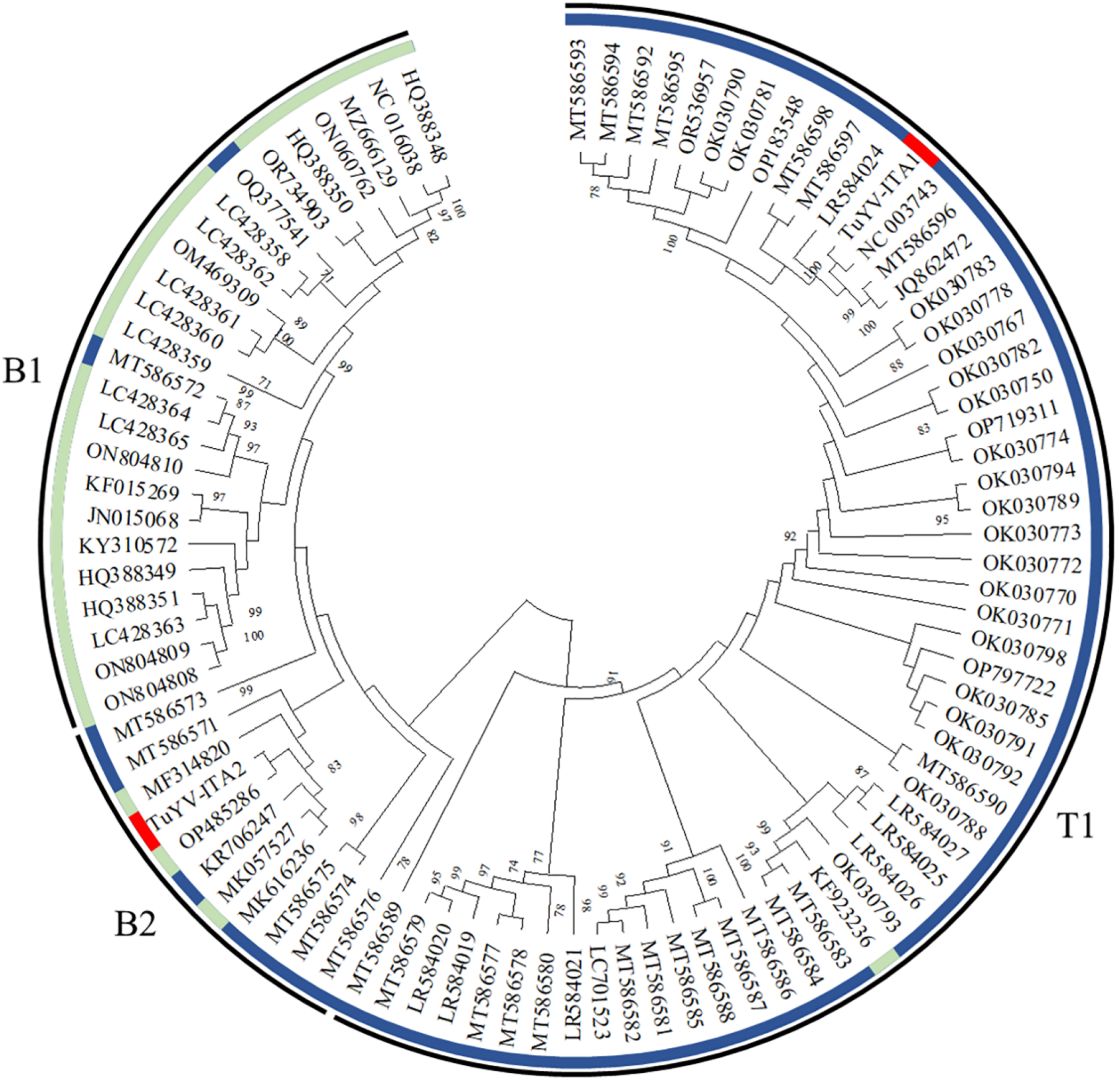
Phylogenetic reconstruction based on the whole genome sequences of turnip yellows virus (TuYV) and brassica yellows virus (BrYV) isolates listed in **Supplementary Table S1** [maximum likelihood method using MEGA X program (Kumar et al., 2018)]. The colored circle identifies the TuYV isolates (blue) and BrYV isolates (light blue). All whole-genome sequences of TuYV and BrYV registered in NCBI were divided into three groups (B1, B2, and T1). The position of the two TuYV isolates identified in the present study are highlighted with red colors. The TuYV-ITA1 isolate (OQ632303) was placed within the T1 group, whereas the TuYV-ITA2 isolate (OQ632304) was clearly placed within the group B2 with ambiguous species distinction.

**Figure 4:**
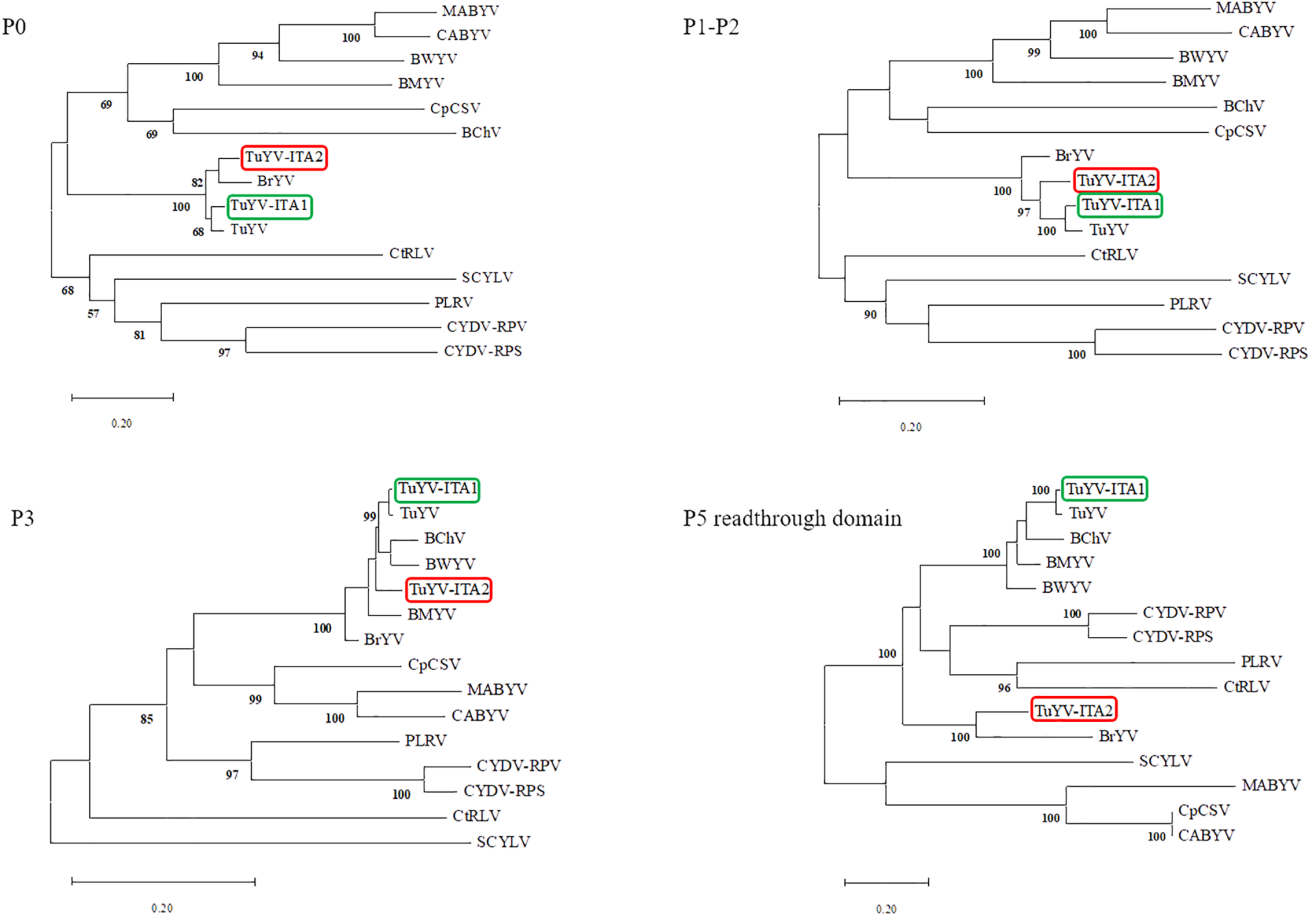
Phylogenetic trees generated from aligned amino acid sequences of the proteins P0, P1–P2, P3, and P5 readthrough domain of different reference poleroviruses species. The phylogeny reconstructions were carried out via the maximum likelihood method and visualized by using the MEGA X program (Kumar et al., 2018), where the bootstrap value was for 1,000 replicates. The following virus sequences (virus abbreviations and accession numbers in parentheses) were obtained from the GenBank database: turnip yellows virus (TuYV, NC_003743), brassica yellows virus (BrYV, NC_016038), beet mild yellowing virus (BMYV, NC_003491), beet western yellows virus (BWYV, NC_004756), carrot red leaf virus (CtRLV, NC_006265), cereal yellow dwarf virus-RPS (CYDV-RPS, NC_002198; CYDV-RPV, NC_004751), sugarcane yellow leaf virus (SCYLV, NC_000874), cucurbit aphid-borne yellows virus (CABYV, NC_003688), beet chlorosis virus (BChV, NC_002766), chickpea chlorotic stunt virus (CpCSV, NC_008249), melon aphid-borne yellows virus (MABYV, NC_010809), and potato leafroll virus (PLRV, NC_001747).

The entire genome of TuYV-ITA1 shared 81.9% nucleotide sequence identity with TuYV-ITA2, with 7.4% of gaps identified between the two sequences, mostly concentrated in the 3’ terminal sequence, as shown in **Supplementary Figure S2**. As a result of the phylogenetic analysis of all whole-genome sequences of TuYV and BrYV registered in NCBI, BrYV was dominant in group B1, and TuYV was dominant in group T1 (**Figure 3**). On the other hand, group B2 contained seven TuYV and three BrYV isolates, making the distinction between the two species ambiguous in this case (**Figure 3**). TuYV-ITA1 was included in group T1 and showed high sequence homology with closely related TuYV isolate FL1 (NC 003743). On the other hand, TuYV-ITA2 was placed within the B2 group in the phylogenetic reconstruction, showing the highest sequence homology of 87% with the BrYV isolate BrYV-HQ (OP485286). The pairwise amino acid identity of TuYV-ITA1 and TuYV-ITA2 coding proteins was 84.7% for P0, 92.3% for P1, 93.8% for P1–P2, 95.5% for P3, 93.7% for P4, 57.7% for P5, and 69.8% for P3–P5. The phylogenetic relationships of TuYV-ITA1 and TuYV-ITA2 with other recognized polerovirus, based on P0, P1–P2, P3, and P3–P5, were consistent with the sequence comparison results (**Figure 4**), suggesting that most of the genetic divergence between the two isolates were concentrated in their 3’-proximal and in particular within the P5 readthrough domain (**Figure 4**).

**Figure 5:**
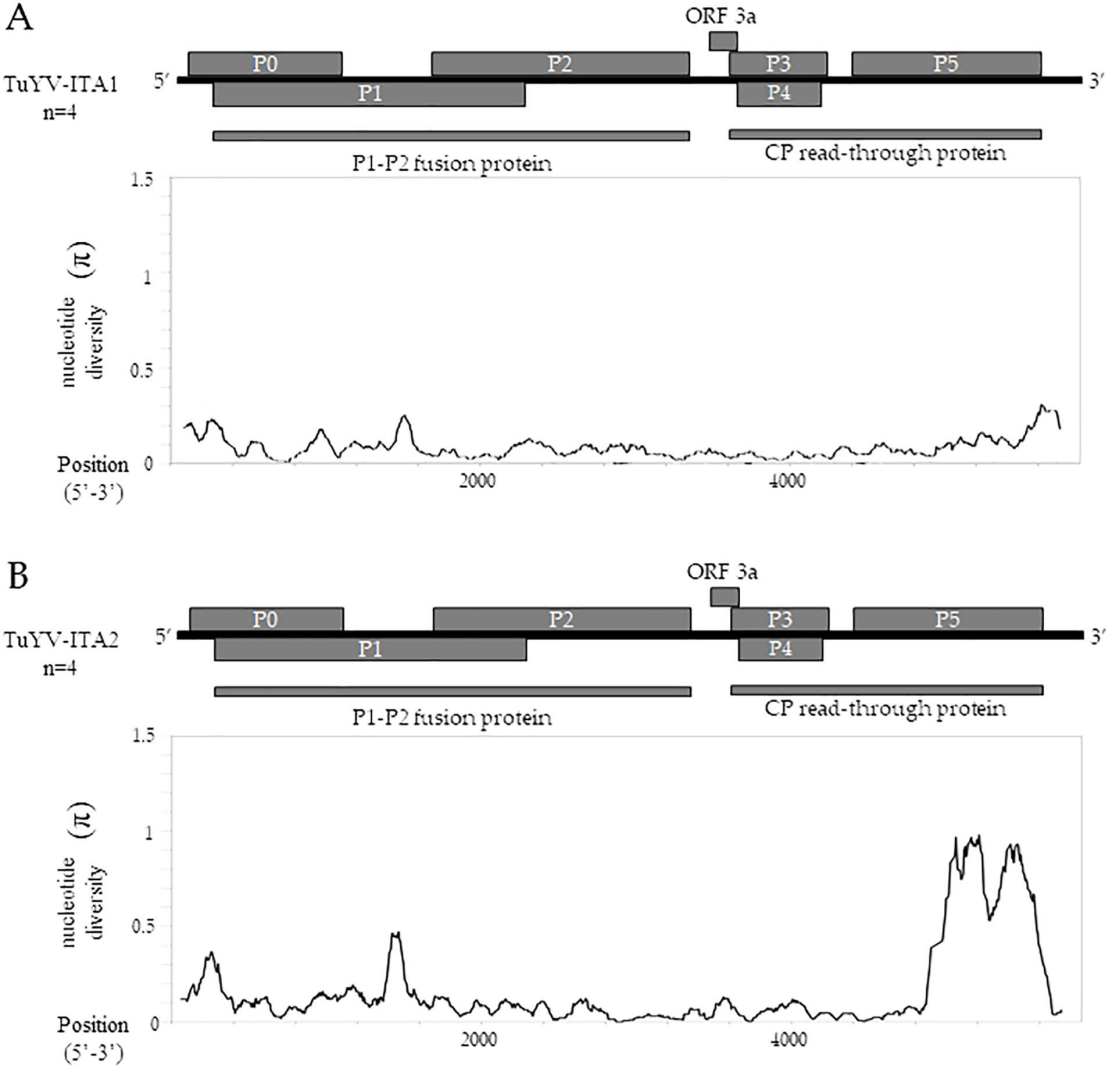
TuYV-ITA1 and TuYV-ITA2 identified in this study were used for nucleotide diversity (π) analysis using the sliding window method implemented in the DNaSP6 software (Rozas et al., 2017). Each virus was aligned with the top four viruses according to BLASTn results, including the following TuYV sequences: X13063, MN497802, OP719311, and MT586598 for the TuYV-ITA1 isolate and MF314820, MK057527, MK616236, and MT586573 for the TuYV-ITA2 isolate. All ORFs are shown for the genome of TuYV-ITA1 **(A)** and TuYV-ITA2 **(B)**.

The sliding window analysis, using a window length of 100 and a step size of 5 bp, showed that TuYV-ITA1 had a relatively low and evenly distributed nucleotide diversity (**Figure 5A**), while TuYV-ITA2 showed an increase in nucleotide diversity (π) in the range between nt positions 4,905 and 5,503 corresponding to the 3’-half of the ORF5 (**Figure 5B**).

Almost in the same genomic region, five out of six algorithms implemented in the RDP4 program (Martin et al., 2015) detected a putative recombination signal (**Table 1**). In particular, a potential recombination event was identified in the TuYV-ITA2 isolate in the genomic region between 4,711 and 5,394 nt, compatible with the region that resulted to be the most divergent within the TuYV-ITA2 genome based on nucleotide diversity analysis obtained with the DNaSP6 program (Rozas et al., 2017) (**Figure 5**). The TuVY-ITA1 isolate was identified as the putative major parental sequence; however, minor parental was labeled as “unknown” (**Table 1**). To get more information about the putative unknown recombinant, BLASTn of the nucleotide sequence between the nucleotides 4,905 and 5,503 was performed. As a result, no sequence showed more than 60% sequence homology, but the annotated viruses were all poleroviruses (**Supplementary Figure S3**).

**Table 1:** Putative recombination events identified by the detection algorithms of the RDP4 software, version 4.101 (Martin et al., 2015), within the whole genome of polerovirus species listed in **Supplementary Table S4** and the whole genome of TuYV-ITA1 and TuYV-ITA2.

Breakpoint position (nt)		Recombinant (accession)	Parental (accession)	
Beginning	End		Major	Minor
4711	5394	OQ632304 (TuYV-ITA2)	OQ632303 (TuYV-ITA1)	Unknown

Detection algorithm (*p*-value)					
RDP	GENECOV	Chimera	MaxChi	BootScan	SiScan
2.65 × 10^-3^	1.06 × 10^-16^	9.55 × 10^-44^	ns	3.04 × 10^-3^	5.12 × 10^-4^

ns: not significant (p-value > 0.01).

### 3.3 Molecular characterization of the CEVd-ITA1 isolate

**Figure 6:**
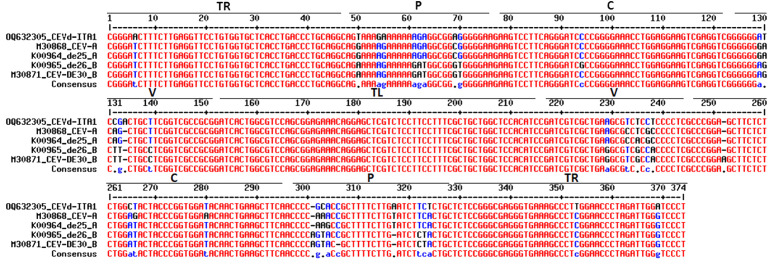
Alignment of CEVD-ITA1 with sequences of class A (CEV and de25) and class B (de26 and DE30) strain as defined by Visvader and Symons (1985). Different domains of the putative secondary structure are indicated: TR, terminal right; P, pathogenicity; C, common; V, variable; and TL, terminal left. The significance of nucleotide colors is based on Corpet (1988): red, high consensus; blue, low consensus; black, neutral; -, deletion.

**Figure 7:**
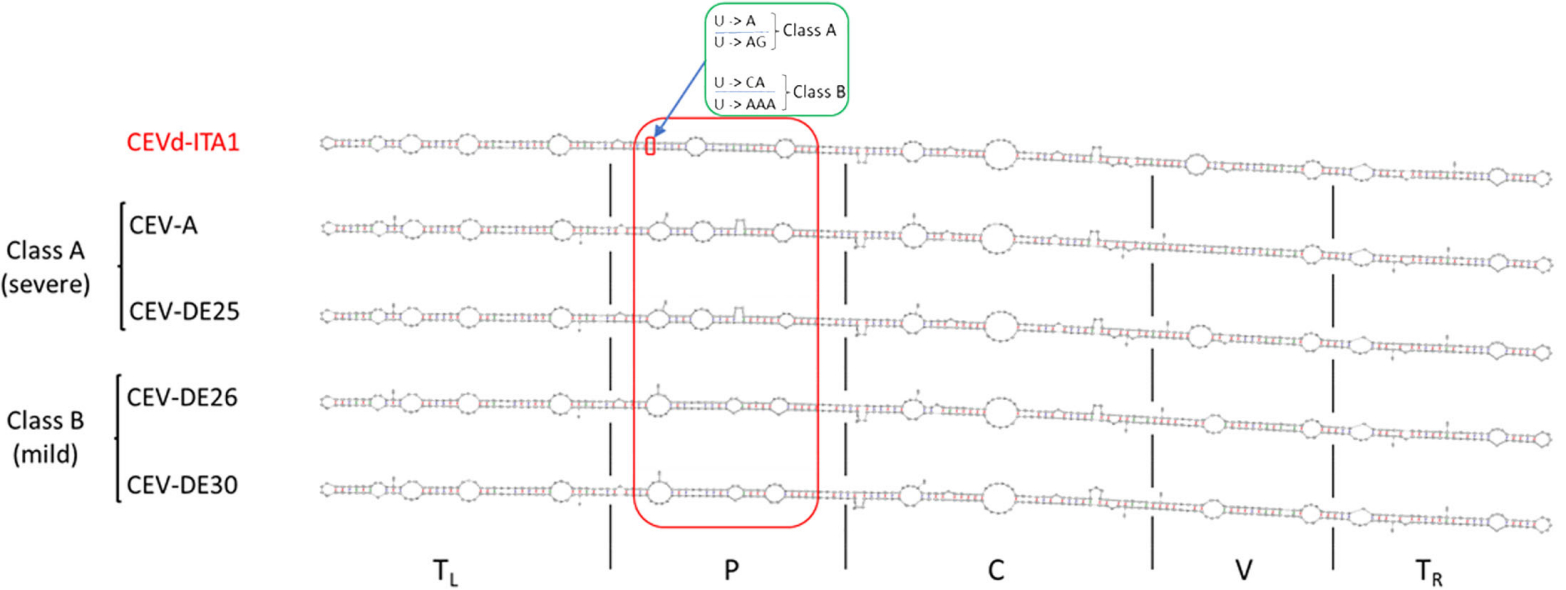
Comparison of the CEVd-ITA1 predicted secondary structure with those of class A (CEV-A and CEV-DE25) and class B (CEVD-DE26 and CEV-DE30) strains, associated respectively to severe and mild symptoms on specific hosts, as defined by Visvader and Symon (1985). Secondary structures of the CEVd sequences were predicted by the MFOLD program (circular version) at 37°C (Zuker, 2003). The red box highlights the different loop positions and their structural differences in the P domain among CEVd_ITA1 and class A and class B strains. The green box highlights the nucleotide difference between CEVd-ITA1 and class A and class B strains correlating with the suppression of a specific loop structure. The U/U paired nucleotides in the CEVd-ITA1 secondary structure (small red box) are substituted with A/AG and CA/AAA in the CEVd class A and B isolates, respectively.

The sequence similarity between CEVd-ITA1 and CEVd sequences retrieved from GenBank ranged from 92.7% to 95.2%, which corresponds to 17–27 in nucleotide differences. The multiple alignment between the CEVd-ITA1 sequence with the reference sequences of the CEVd strains belonging to class A and class B (Gandía et al., 2000) is shown in **Figure 6**. Most of the nucleotide differences were concentrated in the P (pathogenicity) and V (variable) domains. Nevertheless, only nucleotide changes in the P domain correlate with changes in the secondary structure (**Figure 7**). In particular, nucleotide changes concentrated in this domain abolished one loop, which instead is present in the same position of the secondary structure of class A and class B CEVd strains (**Figure 7**). The deviation from the structure of the class A and class B strains can be summarized as follows: change G/A→U at position 50; double change AG→GA at positions 54-55; change C/G→A at position 69; deletion at position 301 (as for class A strains); change A/U→C, at position 303; insertion at position 317, as for class B strain but with the change T→A; and change A→U at position 324 (**Figure 7**).

## 4 Discussion

In the present study, two isolates of TuVY, named TuYV-ITA1 and TuYV-ITA2, and an isolate of CEVd, named CEVd-ITA, have been detected in a mixed infection by HTS analysis from a single plant of *P. americana*, which represents a newly reported host for both virus (Jones et al., 2021) and viroid (Duran-Vila, 2017). In addition, TuYV was detected for the first time in Italy.

The genome-wide phylogenetic relationship between the two isolates TuYV-ITA1 and TuYV-ITA2 and those of TuYV and BrYV obtained from GenBank placed the two isolates in different contexts. While TuYV-ITA1 was placed within group T1, composed of almost all TuYV isolates, the TuYV-ITA2 isolate was placed within the B2 group, consisting of both TuYV and BrYV isolates and thus of ambiguous species distinction (**Figure 3**). This suggested that new genetic traits were possessed by the TuYV-ITA2 isolate. Based on the phylogenetic analysis of P0, P1–P2, P3, and P5 proteins, the two TuYV isolates showed different affinities with the related proteins from other polerovirus (**Figure 4**). While TuYV-ITA1 showed a high phylogenetic relationship with the related proteins of the TuYV reference strain, TuYV-ITA2 showed some affinities with the BrYV and TuYV reference strains for the P0, P1–P2, and P3 proteins and a lower affinity in the case of the P5 protein.

In a previous report in which BrYV was first detected and isolated, BrYV showed a relatively distant relationship only in the P5 readthrough domain with TuYV (Xiang et al., 2011). TuYV-ITA1 showed the same tendency as TuYV isolate FL1 (NC_003743) (**Figure 4**), while TuYV-ITA2 showed the same tendency in the P5 readthrough domain as BrYV isolate BrYV-ABJ (NC_016038) (**Figure 4**).

The ICTV species demarcation criteria for poleroviruses comprise differences in host range, absence of cross-protection, differences in serological detection, and differences in the amino acid sequence of any gene product greater than 10% (King et al., 2011). In our study, the percentages of similarity between TuYV-ITA2 and TuYV-ITA1 proteins meet the ICTV species demarcation criteria only for the P0 and P5 proteins, with a percentage of 84.7% and 57.7%, respectively. Thus, based on the species demarcation criteria, the two viral isolates characterized in the present work should be considered one, TuYV-ITA1, as a type isolate of TuYV while the other, TuYV-ITA2, as a divergent isolate of TuYV. In fact, our results are in agreement with those of Congdon et al. (2023) who found that most of the variations among Australian TuYV isolates concentrated in open reading frame 5 (ORF5), which encodes the readthrough domain (P5) component of the readthrough protein (P3P5), which plays an important role in host adaptation and aphid transmission. Nevertheless, both isolates were identified in *P. americana* which is a previously unreported host for TuYV, and the difference in host range is indicated by the ICTV among the features to be taken into consideration for the definition of a new species of polerovirus. One of the distinctive features of poleroviruses (and of enamoviruses) is the presence of ORF 0 encoding protein 0 (P0), which is a suppressor of gene silencing (La Tourrette et al., 2021). TuYV has a broad host range and shows a complex genetic variability among TuYV isolates. Both of these strictly related factors are probably the reason for its success (Filardo et al., 2021). Some preliminary evidences would indicate that the genetic variability in TuYV ORF 0 regulates the host range of the different TuYV isolates (Newbert, 2016). In polerovirus, protein 0 (P0), the protein genome-linked (VPg), and the coat protein (CP or P3 protein) are involved in virus-vector specificity (Patton et al., 2020), while the read-through domain within CP-RT (P3/P5 domain) participated or contributed to vector transmission, virus movement, and accumulation (Peter et al., 2008). Interestingly, the ORFs coding for the P0 and the read-through domain are the most variable and contain the highest frequency of sites under positive selection (La Tourrette et al., 2021). This is in agreement with our results concerning the TuYV-ITA2 isolate, with the difference that variations identified in the P5 ORF are most probably a consequence of a recombinant event rather than of mutation events. This was confirmed by RDP analysis, while BLAST search using the genomic region between nucleotides 4905 and 5503, the genome region of TuYV-ITA2 with the highest π value, identified sequences with sequence homology lesser than 60%, although all the annotated viruses were poleroviruses (**Supplementary Figure S3**). Therefore, TuYV-ITA2 is presumed to have recombined with an unknown polerovirus that has not yet been characterized.

In polerovirus, both intraspecific and interspecific recombination have been described, occurring in specific hot spots as the intergenic region between P2 and CP or within the RdRp and the 5’ region of P1. Nevertheless, since the read-through domain is a hypervariable genomic region and tolerates mutations better than other polerovirus genomic regions, it has been hypothesized that recombination events are probably likewise tolerated in this region if they do not correlate with a fitness penalty (La Tourrette et al., 2021). This is what we observed in this work where the TuYV-ITA2 isolate showed variability in ORFs 0 and 5, with the latter turning out to be the result of a putative recombination event in this domain.

For some researchers, if a recombination event is suspected, this might indicate that the TuYV isolate belongs to a new species, and thus the whole genome characterization should be done (Filardo et al., 2021). In the case of the TuYV-ITA2 isolate, only two viral proteins do not meet the criteria established by ICTV, P0 and P5, of which the P0 protein seems to accumulate mutations as already reported in the literature, while in the case of the P5 protein, the variability found is most likely the result of a probable recombination event within the ORF 5 with an unknown polerovirus. In this context, it is difficult to definitively conclude whether TuYV-ITA2 is a divergent isolate of TuYV or a new emerging viral species. In all likelihood, based on the data collected, the TuYV-ITA2 isolate represents a variant of TuYV which is evolving to become a new viral species.

In order to adapt to new host or alternative vectors, viruses must maintain a certain ductility in their genome (Rantalainen et al., 2008; Nigam and Garcia-Ruiz, 2020). Nevertheless, to remain in the spreading viral population, viruses must maintain functionality and a high degree of fitness in nature. Consequently, poleroviruses must regulate genomic flexibility and retain fundamental functions. Both intraspecific and interspecific recombination appears to be a strategy successfully used by poleroviruses to achieve the objectives of adaptation to new hosts and/or vectors, maintaining the stability and functionality of their genome (Costa et al., 2019, 2020; La Tourrette et al., 2021).

During HTS analysis, a CEVd sequence was also obtained, indicating that the two TuYV isolates were in a mixed infection with a CEVd isolate in the same *P. americana* plant. The presence of the CEVd-ITA1 isolate was validated by RT-PCR and Sanger sequencing using primers designed on the viroidal sequence obtained by HTS. This is the first report of CEVd in *P. americana*. CEVd-ITA1 showed nucleotide variability mainly within the P domain (pathogenicity) when compared with the severe (class A) and mild (class B) reference strains of CEVd (**Figure 6**). Accordingly, CEVd-ITA1 showed the suppression of a loop in the secondary structure of the P domain (**Figure 7**). Previously, infectivity assays conducted with CEVd chimeric cDNA clones have shown that the changes in the P region are responsible for symptom modulation (Visvader and Symons, 1985). In our case, it is difficult to attribute with certainty whether the evolution of symptoms observed, which evolved from generalized yellowing to dieback and wilting (**Figure 1**), is related to the CEVd infection in the *P. americana* plant, but since in wild species the TuYV infection can be asymptomatic or at least inducing yellowing/redness symptoms, we can cautiously hypothesize that the symptoms observed were induced by the new variant of CEVd or at least by the TuYV-CEVd–plant interaction.

Wild and weed plants can serve as a reservoir for viruses, also ensuring the conservation of their biological–molecular variability in the field. Classic virus diagnostic methods usually detect one or a few viral species and require prior knowledge of the virus to be detected, in particular, for their development and optimization which require the availability of sequence data of the target viruses. Moreover, current knowledge of the population variability of a given virus can be very partial not only for newly identified viruses but also for viruses already established for a long time. As a result, the detection methods developed should not be too generic or polivalent due to the risk of not identifying variants or divergent strains of a given virus. By using HTS approach for the unbiased identification of viral variability in a single *P. americana* plant, we were able to identify two divergent isolates of TuYV in a mixed infection with a novel strain of CEVd. This will allow us to develop and optimize a specific diagnostic tool for the detection, in particular, of the new TuYV variant in order to understand if TuYV and its variant are spreading and may represent a new threat to crops in Italy after this first report. At the same time, further studies will reveal how frequent and important this co-existence is for the spread/infectivity of both pathogens and possible synergisms between poleroviruses and viroids in terms of transmissibility and symptom severity.

## Data Availability

The datasets presented in this study can be found in online repositories. The names of the repository/repositories and accession number(s) can be found in the article/**Supplementary Material**.
