# Supplementary material for: High-throughput sequencing detected a virus–viroid complex in a single pokeweed plant
Source: Front Plant Sci. 2024 Aug 22;15:1435611. doi: 10.3389/fpls.2024.1435611 (PMC11374604; doi:10.3389/fpls.2024.1435611)

Supplementary materials

Table S1 - A BLASTn search against NCBI GenBank performed on the contigs assembled during the *de novo* assembly. The most similar isolates were used as reference sequences for genome reconstruction of TuYV-ITA1 (contig #45), TuYV-ITA2 (contig #87) and CeVD-ITA1 (contig #3781).

**Contig**	**Virus/Isolate**	**Length**	**Identity %**	**Accession No.**
#45	TuYV/FL1	5641 nt	95.28	X13063
#87	TuYV/Anhui	5643 nt	91.80	KR706247
#3781	CEVd/R160510-19	370 nt	94.86	KY110722

Table S2 - Primer pairs used to validate the sequences obtained by HTS analysis, discriminate between TuYV-ITA1 and TuYV-ITA2 (see fig in the text) and for the amplification of CEVd.

Primer pair specificity	Sequence 5′-3′	Size (bp)
**TuYV-ITA1**		
TuYV1-f1	CCTGGTCTGCCAAAACCTCA	282
TuYV1-r1	TTTGGCAAGCTTCTCCGACT	
TuYV1-f2	GAAAACTCCGAGGGATGCGA	629
TuYV1-r2	CAGAACCTTGAGTCGGGGTC	
**TuYV-ITA2**		
TuYV2-f1	TCCGGCAGTCCAAAAGTTGT	604
TuYV2-r1	CTCCCTGCCATCCCAAACAT	
TuYV2-f2	GAGGTTCCGCCCTGAAAAGA	540
TuYV2-r2	GAACCCAGGATTTGCGCTTC	
TuYV2-f1*	CTGGTTCAGACGCAGGTTCT	327
TuYV2-r1*	CCCTGCCATCCCAAACATCT	
TuYV2-f2*	TCTGGTTCAGACGCAGGTTC	310
TuYV2-r2*	CTCCACCCGCTAAGATCGTC	
**CEVd-ITA**		
CEVd-f1	CTTGAGGTTCCTGTGGTGCT	350
CEVd-r1	TAGGGTTCCAAGGGCTTTCAC	
CEVd-f2	CGGAGGGGGAAGAAGTCCT	301
CEVd-r2	CCAATCTAGGGTTCCAAGGGC	

Table S3 - Turnip yellows virus (TuYV) and brassica yellows virus (BrYV) reference isolates retrieved from GenBank and used in this study for the phylogenetic reconstruction.

Virus/Isolate	Accession No.	Host	Country
TuYV/5248	MT586581	*Sinapis arvensis*	Australia
TuYV/C21A	MT586582	*Brassica napus*	Australia
TuYV/P5-8	MT586586	*Pisum sativum*	Australia
TuYV/5509	MT586587	*B. napus*	Australia
TuYV/C2016b	MT586588	*B. napus*	Australia
TuYV/C2016a	MT586585	*B. napus*	Australia
TuYV/-	LR584020	-	Australia
TuYV/MK109	MT586579	*P. sativum*	Australia
TuYV/-	LR584019	-	Australia
TuYV/MK104	MT586578	*B. napus*	Australia
TuYV/MK102	MT586577	*B. napus*	Australia
TuYV/5512b	MT586580	*B. napus*	Australia
TuYV/-	LR584021	-	Australia
TuYV/5752	LC701523	*Allium sativum*	Australia
TuYV/MK113	MT586589	*Lens culinaris*	Australia
TuYV/Ramsey-4	OK030791	*P. sativum*	United Kingdom
TuYV/The Deepings	OK030792	*P. sativum*	United Kingdom
TuYV/Langtoft	OK030771	*P. sativum*	United Kingdom
TuYV/Ramsey-1	OK030798	*P. sativum*	United Kingdom
TuYV/12842	OP797722	*P. sativum*	United Kingdom
TuYV/Chirnside IDT	OK030785	*P. sativum*	United Kingdom
TuYV/MK107	MT586590	*B. napus*	Australia
TuYV/MK103	MT586583	*P. sativum*	Australia
TuYV/MK106	MT586584	*B. napus*	Australia
TuYV/Cambridge-1	OK030793	*P. sativum*	United Kingdom
TuYV/Ramsey-2	OK030788	*P. sativum*	United Kingdom
TuYV/-	LR584024	-	Australia
TuYV/-	LR584025	-	Australia
TuYV/-	LR584026	-	Australia
TuYV/-	LR584027	-	Australia
TuYV/Chatteris	OK030770	*P. sativum*	United Kingdom
TuYV/Louth	OK030772	*P. sativum*	United Kingdom
TuYV/East Anglia	OK030794	*P. sativum*	United Kingdom
TuYV/March	OK030789	*P. sativum*	United Kingdom
TuYV/Karpalund	OP719311	*B. napus* subsp. *napus*	Sweden
TuYV/MKT WGN Symp	OK030774	*P. sativum*	United Kingdom
TuYV/Market Weighton	OK030773	*P. sativum*	United Kingdom
TuYV/LGN Symp	OK030783	*P. sativum*	United Kingdom
TuYV/WNFT Symp	OK030778	*P. sativum*	United Kingdom
TuYV/Br12	MT586598	*Beta vulgaris*	England
TuYV/C20A	MT586597	*B. napus*	Australia
TuYV/Langton	OK030782	*P. sativum*	United Kingdom
TuYV/Market Weighton	OK030750	*P. sativum*	United Kingdom
TuYV/5512a	MT586593	*B. napus*	Australia

TuYV/5514a	MT586594	*B. napus*	USA
TuYV/5513	MT586592	*B. napus*	Australia
TuYV/Cil_Cl	OR536957	*Coriandrum sativum*	Chile
TuYV/Ramsey-3	OK030790	*P. sativum*	United Kingdom
TuYV/MK105	MT586595	*B. napus*	Australia
TuYV/Goettingen	OP183548	*B. napus*	Germany
TuYV/E Symp	OK030781	*P. sativum*	United Kingdom
TuYV/Canterbury	OK030767	*P. sativum*	United Kingdom
TuYV/5514b	MT586576	*B. napus*	USA
TuYV/L31-4	MT586574	*L. culinaris*	Australia
TuYV/P6-2	MT586575	*P. sativum*	Australia
TuYV/MK111	MT586573	*C. arietinum*	Australia
TuYV/5594	OQ377541	*B. rapa* subsp. *pekinensis*	Australia
TuYV/1740	MT586571	*Cicer arietinum*	Australia
TuYV/5510	MT586572	*C. arietinum*	Australia
TuYV/HN/tobacco/2017	MK616236	*Nicotiana tabacum*	China
TuYV/ Anhui	KR706247	*N. tabacum*	China
TuYV/FL1	NC_003743	*Lactuca sativa*	France
TuYV/5511	MT586596	*C. arietinum*	Australia
TuYV/WA-1	JQ862472	*Diuris* sp.	Australia
BrYV/Mibao	MZ666129	*Fragaria* sp.	China
BrYV/BrYV-HY	ON060762	*Fragaria* sp.	China
BrYV/BrYV-ABJ	NC_016038	*B. napus* var. *napobrassica*	China
BrYV/BrYV-ABJ	HQ388348	*B. napus* var. *napobrassica*	China
BrYV/P15	OR734903	*Arachis hypogea*	South Korea
BrYV/BrYV-AJS	HQ388350	*B. campestris*	China
BrYV/WN1	LC428359	*Sinapis alba*	Japan
BrYV/CD9	LC428362	*B. oleracea* var. *capitata*	Japan
BrYV/CC1	LC428358	*B. rapa* subsp. *pekinensis*	Japan
BrYV7TO3	LC428360	*B. rapa* subsp. *rapa*	Japan
BrYV/NAP	LC428361	*B. napus*	Japan
BrYV/BrYV-Tas	OM469309	*Raphanus raphanistrum*	Australia
BrYV/BrYV-lnc	ON804808	*B. napus*	China
BrYV/BrYV-814NJLH	ON804809	*B. napus*	China
BrYV/BrYV-BBJ	HQ388349	*B. napus* var. *napobrassica*	China
BrYV/R3b	LC428363	*R. sativus*	Japan
BrYV/ BrYV-BJS	HQ388351	*B. campestris*	China
BrYV/China	KY310572	*B. napus*	China
BrYV/RT8	LC428364	*R. sativus*	Japan
BrYV/R40	LC428365	*R. sativus*	Japan
BrYV/BrYV-NJ13	ON804810	*B. napus*	China
BrYV/CR	JN015068	*R. raphanistrum*	China
BrYV/CC	KF015269	*B. rapa* supsp. *pekinensis*	China
BrYV/CC	KF923236	*B. rapa*	South Korea
BrYV/BrYV-NtabQJ	MK057527	*N. tabacum*	China
BrYV/Anhui	MF314820	*N. tabacum*	China
BrYV/BrYV-HQ	OP485286	*Scutellaria baicalensis*	China

Table S4 - Selected member species of poleroviruses used in this study.

Virus	Acronym	Accession
Turnip yellows virus	TuYV	NC_003743
Brassica yellows virus	BrYV	NC_016038
Beet mild yellowing virus	BMYV	NC_003491
Beet western yellows virus	BWYV	NC_004756
Carrot red leaf virus	CtRLV	NC_006265
Cereal yellow dwarf virus-RPS	CYDV-RPS	NC_002198
Cereal yellow dwarf virus-RPV	CYDV-RPV	NC_004751
Sugarcane yellow leaf virus	SCYLV	NC_000874
Cucurbit aphid-borne yellows virus	CABYV	NC_003688
Beet chlorosis virus	BChV	NC_002766
Chickpea chlorotic stunt virus	CpCSV	NC_008249
Melon aphid-borne yellows virus	MABYV	NC_010809
Potato leafroll virus	PLRV	NC_001747

Figure S1 - The read counts of TuYV-ITA1, TuYV-ITA2, and CEVd isolates identified in this study were quantified by remapping to their respective assembled genomes. Each virus was depicted with a distinct color in the pie chart showed.

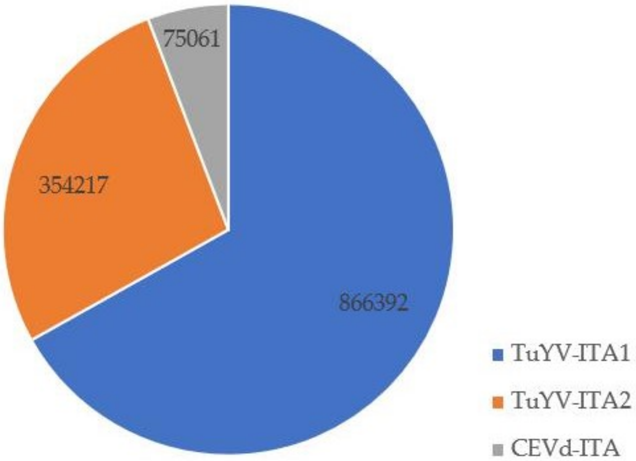

Figure S2. Alignment between TuYV-ITA1 and TuYV-ITA2 with the indication of the position of the primers used to discriminate the two isolates (see Figure 2 and Table S3). In the alignment, the stars correspond to equal nucleotides between the two sequences. The position of the specific primers for TuYV-ITA1 and TuYV-ITA2 are indicated and their sequence highlighted in yellow, while common nucleotide sequence between two overlapping or continuous primers are highlighted in green.

```
OQ632303_TuYV-ITA1      CCAGGAGGGAATCCTTAGTTGATGCAATTTCTCGCTCACGATAACTTTCACACCTTGCAA 60
OQ632304_TuYV-ITA2      CCAGGAGGGAATCCTAAGTAGATGCAATTTGTTGCTCACGATAACTTTCACACTCTAGAA 60
                        *************** *** ********** * ********************  *  **

OQ632303_TuYV-ITA1      GTTAAGAAAGTTCGATTCCTCCACTCTCAACAAGAAGCGTTTCTTTTAGCAGGTTTATTG 120
OQ632304_TuYV-ITA2      GTCAGGAAAGTCAGATACCTCCATCCACAACAAGTATTGTTTCTTTTAGCAGGTTTATTG 120
                        ** * ******  *** ******  * ******* *  **********************

OQ632303_TuYV-ITA1      CTTAACATAAAACAATTTGTACAAGCAATCAAAGAGCACAACAATGAATTCAAAATTGAC 180
OQ632304_TuYV-ITA2      CTTAACATCAAACGATTTGTAAAAGCGATCCAAGAGCGTAACAATGAGTTCAAAACTGAT 180
                        ******** **** ******* **** *** ******  ******** ******* ***

OQ632303_TuYV-ITA1      GTTTTTCTTCGCTCTTTGCTTTATCAGCTTCCTTTCCACATCGGAAGCTGCTTCCACGAT 240
OQ632304_TuYV-ITA2      GTTTTTCTTCGCTCTTTGCTTTATCAGCTTCCTTTCCACATCGGAAGCTGCTTCCACGAT 240
                        ************************************************************

OQ632303_TuYV-ITA1      GCTCTTCGAGAGCACATACCTGCCCCTGAACCAGAGCTGTGCGCCTGGTTTTCTTTACAA 300
OQ632304_TuYV-ITA2      GCTCTTCGAGAGCACATACCTGCCCCTGAACCAGAGCTATGCGCCCGGTTTTCTTTACAA 300
                        ************************************** ****** **************

OQ632303_TuYV-ITA1      ACGGGATACGCTCCCGCCTCCACTACAGGCTGTGCTAACCTACACGTGCCCGGAACCAAG 360
OQ632304_TuYV-ITA2      ACGGGATATGCTCCCGCCTCCACCTCAGGCCGTATTGACTTACACGTGCCAGGAACCCAG 360
                        ******** **************  ***** **  * ** ********** ****** **

OQ632303_TuYV-ITA1      ACCTCTCGCAGAAGAATCATACAACGATCTTTTGCGAGCGATTTCTCAGAAAAGCTCAAG 420
OQ632304_TuYV-ITA2      ACCTCTCGTAGAAGAATCATACAACGATCTTTTGCGAGCAATTTCTCAGAAAAGCTCAAG 420
                        ******** ****************************** ********************

OQ632303_TuYV-ITA1      CGATTTCCAGAATGCCTATTCGTTAGCCTTGAGTTTTTCCAGCGACTTCTATCAACATGG 480
OQ632304_TuYV-ITA2      CGATTTCCAGAATGCTTATTCGTTAGCCTTGAGTTTTTCCAGCGATTTCTGTCAATCTGG 480
                        *************** ***************************** **** ****  ***

OQ632303_TuYV-ITA1      ACTAAAGACATTGAAAGACGTGTCCTTTCTGGCTGTCGAGAAATTCCTTTGGGGTCTGAC 540
OQ632304_TuYV-ITA2      ACTAGAGACGCTGAAAGACGCCTCTTTTCTGGCTGTCGAGAAATTCCTGTGGGGTCTCAC 540
                        **** ****  *********  ** *********************** ******** **

OQ632303_TuYV-ITA1      ACGCTTATGGAGCTCGTTAATCTTGGCGAGCTTCTCCGCGTTATGGTGGTTGGTGAGCAA 600
OQ632304_TuYV-ITA2      ACGCTTGTGGAGCTCACTCATATTGGCGAGCTTCTCCATGATATGGTGGTTGGTGAGAAA 600
                        ****** ********  * ** ***************  * **************** **

OQ632303_TuYV-ITA1      TTTCACAACTCCCGTCTTCTGTCTCGCCTTGCTGTACACTGTTACAAGATTTATGGTGAA 660
OQ632304_TuYV-ITA2      TTTCACGACTCCCGTCTTCTGTCTCGCCTTGCTGTACACTGTTACAAGATTTATGGCGAA 660
                        ****** ************************************************* ***

OQ632303_TuYV-ITA1      GACGGTTTCATTTCTTTTTGGAGGATTGCCAATCTGGATCATTTCGATTGCTTTCTCACT 720
OQ632304_TuYV-ITA2      GATGGTTTCATTTCTTTTTGGAGGATTGCCAATTTGGATCATCTCGATTATTTTCTCACT 720
```

```
                             ** ****************************** ******** ******  *********

OQ632303_TuYV-ITA1      CCTGAAGAAATCCTTTTCAGCTCTTCGGTCTACACCGAAATGTTTGTATGAGAAGGCTAT 780
OQ632304_TuYV-ITA2      CCTGAAGAAATCCTTTTCAGCTCTTCGGTCTACGCCGAAATGTTTGTATGAAAAGGCTAT 780
                        ********************************* ***************** ********

OQ632303_TuYV-ITA1      AGACGGTTTCAAGAGTTTCACTATCCCGCAGAGTCCTCCAAAATCTTGCGTGATTCCTAT 840
OQ632304_TuYV-ITA2      AGACGGTTTTAAGAGTTTTACTATCCCGCAAAGCCCACCCAAATCTTGCGTGATTCCCAT 840
                        ********* ******** *********** ** ** ** ***************** **

OQ632303_TuYV-ITA1      CACCCACGCAAGCGGAAACCACGCTGGTTATGCCAGTTGTGTCAAGCTGTACAACGGAGA 900
OQ632304_TuYV-ITA2      CACCCACGCAAGCGGAAACCACGCTGGTTATGCCAGCTGTGTGAAGCTGTACAATGGCGA 900
                        ************************************ ***** *********** ** **

OQ632303_TuYV-ITA1      AAATGCTTTAATGACGGCGACCCACGTTCTACGTGATTGTCCCAATGCCGTGGCTGTTTC 960
OQ632304_TuYV-ITA2      GAATGCTTTGATGACGGCGACCCACGTTTTACGTGACTGCCCCAATGCTGTAGCTGTTTC 960
                         ******** ***************** ******* ** ******** ** ********

OQ632303_TuYV-ITA1      CGCTAAAGGGCTCAAGACTCGGATTCCACTCGCTGAATTCAAAACAATCGCGAAATCCGA 1020
OQ632304_TuYV-ITA2      CGCCAAAGGGCTCAAAACTCGGATCCCACTTGCTGAATTTAAAACGATTGCGAGATCCGA 1020
                        *** *********** ******** ***** ******** ***** ** **** ******

OQ632303_TuYV-ITA1      CAAAGGTGATGTTACCCTCCTTCGCGGCCCCCCCAATTGGGAAGGACTGTTAGGCTGTAA 1080
OQ632304_TuYV-ITA2      CAAAGGTGATGTAACCCTCCTCCGTGGCCCCCCTAATTGGGAAGGATTGTTGGGCTGCAA 1080
                        ************ ******** ** ******** ************ **** ***** **

OQ632303_TuYV-ITA1      AGCGGCCAACATCATAACTGCTGCTAACCTAGCGAAATGCAAAGCAACCATATACTCTTT 1140
OQ632304_TuYV-ITA2      AGCGGCCAACGTCATAACGGCCGCCAACCTAGCGAAATGCAAAGCGTCCATATACTCTTT 1140
                        ********** ******* ** ** ********************  *************

OQ632303_TuYV-ITA1      TGACAGAGATGGCTGGGTTGGCAGTTATGCCGAGATTGTGGGCTCAGAAGGCACGGATGT 1200
OQ632304_TuYV-ITA2      CGAGAGAGACGGCTGGGTTAGCGGCTATGCTGAGATTGTAGGCTCTGATGGCACCGATGT 1200
                         ** ***** ********* ** * ***** ******** ***** ** ***** *****

OQ632303_TuYV-ITA1      TATGGTTCTGAGCCACACGGAAGGAGGACACTCCGGAAGCCCCTATTTCAATGGTAAAAC 1260
OQ632304_TuYV-ITA2      TATGGTACTGAGCCACACGGAAGGAGGACACTCCGGAAGCCCCTACTTTAATGGCAAAAC 1260
                        ****** ************************************** ** ***** *****

OQ632303_TuYV-ITA1      CATTTTGGGGGTTCACTCAGGTGCCAGTGCTACTGGAAATTACAATTTAATGGCACCAAT 1320
OQ632304_TuYV-ITA2      TATTTTGGGGGTCCACTCGGGTGCCAGTGCTACTGGAAACTACAACCTAATGGCACCAAT 1320
                         *********** ***** ******************** *****  *************

OQ632303_TuYV-ITA1      CCCATCCCTCCCCGGGCTTACCAGTCCGACTTATGTGTTCGAAACCACCGCACCACAAGG 1380
OQ632304_TuYV-ITA2      CCCATCCATCCCCGGTCTTACAAGCCCCACTTATGTGTTTGAAACCACCGCACCACAAGG 1380
                        ******* ******* ***** ** ** *********** ********************

OQ632303_TuYV-ITA1      AAGAGTTTTCGCACAAGAAGATATCGCCGAAATCGAAGGCCTTTATGCGCAAGTAATGAA 1440
OQ632304_TuYV-ITA2      AAGAGTTTTCGCACAAGAAGATATCGCCGAAATCGAAGGCCTTTATGCGCAAGTAATGAA 1440
                        ************************************************************

OQ632303_TuYV-ITA1      AAGAGTTCAACAAACGGAAGATTTCAAACCCAAAACTGGAAAGTACTGGGGTGATATGGA 1500
OQ632304_TuYV-ITA2      AAGAGTTCAACAAACGGAAGATTTCAAGTCCAAAACTGGAAAGAACTGGGCTGATGAGGA 1500
                        ***************************  ************** ****** ****  ***

OQ632303_TuYV-ITA1      GGATGATGAAGACATTTTCTTCGAAAGCAAAGAAGATCTGTCGGGAAACGGAGTGCGCGG 1560
OQ632304_TuYV-ITA2      T---GATGATGATATCTTCTTCGAAAGCAAAGAAGATTTTTCGGGAAACGGAGTGCGCGG 1557
                            ***** ** ** ********************* * ********************

OQ632303_TuYV-ITA1      CACCGTCCGCGGAACAAACGGAGAAGGCAGCTCCACCCCAAAGACAAGCAACGTCGATGG 1620
OQ632304_TuYV-ITA2      CGCCGACCGCGAAACAAACGGCGAAGGCAGCTCCACCCCAAAGACAAACAACGTCGATGG 1617
```

```
                        * *** ***** ********* ************************* ************

OQ632303_TuYV-ITA1      GAAAGAGATGATGGAGAAAATAATCTCATCTCTAGTGGGAAAGATAAATCTCGAGAACAT 1680
OQ632304_TuYV-ITA2      GAAAGAGATGATGGAGAAAATAATCTCGTCTCTAGTGGGAAAGATAAATCTCGAGAACAT 1677
                        *************************** ********************************


OQ632303_TuYV-ITA1      CGAGAGGAAAGTGATAGAGGAGATCTCCGCGAAAGCGATGAAAACTCCGAAATCTCGCCG 1740
OQ632304_TuYV-ITA2      CGAGAAGAAAGTGGTCGAACAGATCTCCGCGAAAGCGATGAAGAAACCAAGACCCAGCCG 1737
                        ***** ******* * **  ********************** *  ** * * *  ****

OQ632303_TuYV-ITA1      CAGAAGAGCCCCAAAGAAACAGCCGGAGAGTTCGAAAGATACTTCTCCTCGCTCTACAAT 1800
OQ632304_TuYV-ITA2      CAGAAGAGCCCCAAAGAAACAGCCGGAGAGTTCCAAAGATACTTCTCCTCGCTCTACGAC 1797
                        ********************************* *********************** *

OQ632303_TuYV-ITA1      TGGGAAGTACCAACCTCCCCACGTGAGGTCCCCGGCTTCCGTCACTGCGGCAAGCTGCCC 1860
OQ632304_TuYV-ITA2      TGGGAAGTACCAACCTCCCCACGTGAGGTCTCCGGCTTCCGCCACTGCGGCAAGCTGCCC 1857
                        ****************************** ********** ******************

OQ632303_TuYV-ITA1      CAATACTACCACCCCAAGCAAAAAGAAGAATCCAGCTGGGGGAAGACCCTCGTCGGGAAC 1920
OQ632304_TuYV-ITA2      CAATACTACCACCCCAAGCAAAAAGAAGAATCAAGCTGGGGGAAAACCCTCGTCGGGAAC 1917
                        ******************************** *********** ***************

OQ632303_TuYV-ITA1      CATCCCGCGTTGGGTGAGAAAACAAGCGGCTTCGGCTGGCCCAAGTTCGGCCCCGAAGCA 1980
OQ632304_TuYV-ITA2      CATCCCGCGTTGGGTGAGAAAACAAGCGGCTTCGGCTGGCCCAAGTTCGGTCCCGAAGCA 1977
                        ************************************************** *********

OQ632303_TuYV-ITA1      GAACTGAAGAGCCTGCGTCTGCAGGCTTCAAGGTGGCTGGAACGCGCCCAGTCCGCAGAA 2040
OQ632304_TuYV-ITA2      GAATTGAAAAGCCTGCGTCTGCAGGCCTCAAGGTGGCTGGAACGCGCCCAGTCCGCAGAA 2037
                        *** **** ***************** *********************************

OQ632303_TuYV-ITA1      ATACCCTCTGACGCTGAGAGGGAGCGTGTGATTCAAAAGACCGCAGATGTTTACCATCCC 2100
OQ632304_TuYV-ITA2      ATACCCTCTGACGCTGAGAGGGAGCGTGTGATTAAAAAGACCGCGGATGTTTACCATTCT 2097
                        ********************************* ********** ************ *

OQ632303_TuYV-ITA1      TGCCAAACCAATGGACCTGCGGCAACCCGAGGAGGGACACTGACCTGGAACAACTTCATG 2160
OQ632304_TuYV-ITA2      TGCCAAACTAATGGACCCGCGGCGACCCGAGGAGGAGCACTGACCTGGAATAATTTTATG 2157
                        ******** ******** ***** ***********  ************* ** ** ***

OQ632303_TuYV-ITA1      ATAGATTTCAAACAGGCTGTGTTCTCGCTGGAGTTCGACGCCGGAATAGGCGTCCCCTAT 2220
OQ632304_TuYV-ITA2      ATTGATTTCAAACAGGCTGTGTTCTCGCTTGAGTTCGATGCTGGAATAGGCGTCCCCTAT 2217
                        ** ************************** ******** ** ******************

OQ632303_TuYV-ITA1      ATTGCTTATGGCAAGCCCACGCATCGTGGGTGGGTTGAAGATCAGAAACTCCTCCCAGTT 2280
OQ632304_TuYV-ITA2      ATTGCCTATGGCAAGCCCACGCATCGTGGGTGGGTTGAAGACCAGACACTCCTCCCAGTC 2277
                        ***** *********************************** **** ************

OQ632303_TuYV-ITA1      CTAGCTCAATTGACCTTCATCCGATTACAGAAGATGTTGGAGGTCAATTTCGAAGATATG 2340
OQ632304_TuYV-ITA2      CTAGCTCGATTGACCTTCATCCGACTACAGAAGATGTTGGAGGTTAATTTTGAAGATATG 2337
                        ******* **************** ******************* ***** *********

OQ632303_TuYV-ITA1      GAACCAGAGGAGCTGATCCGGAACGGTTTGTGCGACCCCATCCGATTATTCGTGAAGGGT 2400
OQ632304_TuYV-ITA2      GGACCTGAGGAGCTGGTTCAGAACGGCTTGTGCGATCCAATCAGATTGTTCGTAAAAGGT 2397
                        * *** ********* * * ****** ******** ** *** **** ***** ** ***

OQ632303_TuYV-ITA1      GAGCCGCACAAGCAAGCGAAGCTCGATGAAGGCCGCTACCGCCTCATAATGAGCGTTTCC 2460
OQ632304_TuYV-ITA2      GAACCCCACAAGCAAGCGAAGCTCGATGAAGGCCGCTACCGCCTCATTATGAGTGTTTCC 2457
                        ** ** ***************************************** ***** ******

OQ632303_TuYV-ITA1      CTCGTGGATCAACTGGTAGCCCGGGTTCTGTTTCAAAATCAGAACAAGCGGGAAATTGCC 2520
OQ632304_TuYV-ITA2      CTCGTGGATCAATTGGTGGCCCGGGTTCTGTTTCAAAATCAGAACAAGCGGGAAATCGCC 2517
```

```
                          ************ **** ************************************** ***

OQ632303_TuYV-ITA1        CTGTGGAGGGCCATCCCCAGTAAACCCGGTTTTGGCTTGTCCACGGACGAGCAAGTGCTG 2580
OQ632304_TuYV-ITA2        TTATGGAGGGCTATCCCCAGTAAACCCGGTTTTGGCTTGTCCACGGATGAGCAAGTGCTG 2577
                           * ******** *********************************** ************


OQ632303_TuYV-ITA1        GACTTCGTGGAAAGTCTGGCCCGTCAAGTAGGCACCACTACGACAGAGGTGGTTGCCAAT 2640
OQ632304_TuYV-ITA2        GACTTTGTAAAAGGTCTGGCCCGTCAAGTAGGCACTACAACAGAAGATGTGGTTGCCAAT 2637
                          ***** **  ** ********************** ** **   *** ************

OQ632303_TuYV-ITA1        TGGAAGAATTACTTGACGCCCACGGATTGCTCCGGCTTTGACTGGAGTGTTGCGGATTGG 2700
OQ632304_TuYV-ITA2        TGGAACAATTACTTGACGCCCACGGATTGCTCTGGTTTTGACTGGAGTGTTGCGGATTGG 2697
                          ***** ************************** ** ************************

OQ632303_TuYV-ITA1        ATGCTTCACGACGACATGATCGTCCGCAATAGACTTACCATCGACCTCAACCCCGCTACA 2760
OQ632304_TuYV-ITA2        ATGCTTCATGATGATATGATCGTCCGCAACAGACTTACCATCGACCTCAACCCCGTAACA 2757
                          ******** ** ** ************** *************************  ***

OQ632303_TuYV-ITA1        GAAAGATTAAGATCCTGCTGGTTGAGGTGCATTTCGAACTCAGTATTGTGCCTGAGTGAT 2820
OQ632304_TuYV-ITA2        GAAAGATTAAGATCCTGTTGGTTGAGATGTATTTCAAACTCAGTTTTATGCTTGAGCGAT 2817
                          ***************** ******** ** ***** ******** ** *** **** ***

OQ632303_TuYV-ITA1        GGTACCCTTTTAGCCCAAACTCATCCGGGCGTTCAGAAGAGTGGGAGCTACAACACATCA 2880
OQ632304_TuYV-ITA2        GGTACCCTTTTAGCCCAAACCCATCCGGGCGTTCAGAAAAGTGGGAGCTACAACACATCA 2877
                          ******************** ***************** *********************

OQ632303_TuYV-ITA1        AGCTCCAACTCCCGGATCCGAGTTATGGCCGCCTTCCACACAGGTGCCATTTGGGCTATG 2940
OQ632304_TuYV-ITA2        AGCTCCAACTCCCGGATCAGAGTTATGGCCGCCTTTCACACAGGTGCTGCCTGGGCTATG 2937
                          ****************** **************** ***********    *********

OQ632303_TuYV-ITA1        GCGATGGGTGATGACGCCCTCGAGTCCAATCCCGCTGACCTAGCAGCGTACAAGAAACTA 3000
OQ632304_TuYV-ITA2        GCGATGGGTGACGACGCCCTCGAGTCCAATCCCGCTGACCTAGCAGCGTACAAAAGACTA 2997
                          *********** ***************************************** * ****

OQ632303_TuYV-ITA1        GGCTTCAAGGTTGAGGTTTCCGGACAACTGGAATTCTGCTCTCACATTTTTAGAGCGCCG 3060
OQ632304_TuYV-ITA2        GGATTCAAGGTAGAGGTTTCCGAACAACTGGAATTCTGCTCTCACATTTTTAGAGCGCCG 3057
                          ** ******** ********** *************************************

OQ632303_TuYV-ITA1        GACCTCGCCCTCCCTGTGAACGAAAATAAGATGATCTACAAATTGATCTATGGCTATAAT 3120
OQ632304_TuYV-ITA2        GACCTCGCCCTCCCTGTGAATGAAAACAAAATGATCTACAAGTTGATCCATGGCTACAAC 3117
                          ******************** ***** ** *********** ****** ******* ** 

OQ632303_TuYV-ITA1        CCAGGGAGCGGAAACGCTGAGGTAGTTTCAAACTACTTGGCCGCTTGTTTCTCAGTTCTG 3180
OQ632304_TuYV-ITA2        CCAGGGAGCGGAAACGCCGAGGTAGTTTCCAACTACTTAGCCGCCTGTTTCTCAGTTCTG 3177
                          ***************** *********** ******** ***** ***************

OQ632303_TuYV-ITA1        AACGAGTTGCGGCATGATCCAGCGTCCGTTGAACTTCTTTACTCGTGGTTAGTCGATCCG 3240
OQ632304_TuYV-ITA2        AACGAGTTGCGGCATGATCCGGCGTCCGTTGAACTTCTTTACTCGTGGTTAGTTGACCCA 3237
                          ******************** ******************************** ** ** 

OQ632303_TuYV-ITA1        GTGCTACCACAAAAGATACCAGGAGAGTAAAGAAGA-AGAGAGTCAGCTTACATTGAAAT 3299
OQ632304_TuYV-ITA2        GTGCTACCACAAAAGATATCCAGAGAGTAAAGAAGCAAACAAGTCAGCTTACATTGAAAT 3297
                          ****************** *  *************  *   *******************

OQ632303_TuYV-ITA1        TTTAAAGAGGTTTCTGCAACAGTAAGAGACTTAAGCAAACCCAATTAAAGATACAACGGA 3359
OQ632304_TuYV-ITA2        TCTAAAGAGGTTTCTGCAACAGTAAGAGACTTAAGCAAACCCACTGAAAGATACAACGGA 3357
                          * ***************************************** * **************

OQ632303_TuYV-ITA1        TTACAAATTCCTAGCAGGTTTCGCCGCAGGCTTCGTTTCATCGATACCAATATCCGTAAT 3419
OQ632304_TuYV-ITA2        TTACAAATTCCTAGCAGGCTTTGCCGCAGGCTTCGTTTCATCGATACCAATATCCGTGAT 3417
```

```
                        ****************** ** *********************************** **

OQ632303_TuYV-ITA1      CAGTATCTATTTCATCTACCTAAGAATCTCCAAACACGTACGCGAAATCGTTAATGAATA 3479
OQ632304_TuYV-ITA2      CAGTATCTATTTCATCTACCTAAGAATCTCCAAACACGTGCGCGAAATCGTTAATGAATA 3477
                        *************************************** ********************


OQ632303_TuYV-ITA1      CGGTCGTGGGTAGGAGAACAATCAATGGAAGAAGACGACCACGCAGGCAAACACGACGCG 3539
OQ632304_TuYV-ITA2      CGGTCGTGGGTAGGAGAACAATAAATGGAAGAAGACGACCACGCAGGCAAACACGACGCG 3537
                        ********************** *************************************

OQ632303_TuYV-ITA1      CTCAGCGCTCTCAGCCAGTGGTTGTGGTCCAAACCTCTCGGGCAACACAACGCCGACCTA 3599
OQ632304_TuYV-ITA2      CTCAGCGCTCTCAGCCAGTGGTTGTGGTCCAAACCTCTCGGACAACACAACGCCGACCTA 3597
                        ***************************************** ******************

OQ632303_TuYV-ITA1      GACGACGACGAAGAGGTAACAACCGGACAGGAAGAACTGTTCCTACCAGAGGAGCAGGTT 3659
OQ632304_TuYV-ITA2      GACGACGACGAAGAGGTAACAACCGGACAGGAAGAACTGTTTCTACCAGAGGAGCAGGTT 3657
                        ***************************************** ******************

OQ632303_TuYV-ITA1      CGAGCGAGACATTTGTTTTCTCAAAAGATAATCTCGCGGGAAGTTCCAGCGGAGCAATCA 3719
OQ632304_TuYV-ITA2      CGAGCGAGACATTTGTTTTCTCAAAAGACAATCTCGCGGGAAGTTCCAGCGGAGCAATCA 3717
                        **************************** *******************************

OQ632303_TuYV-ITA1      CGTTCGGGCCGAGTCTATCAGACTGCCCGGCATTCTCTAATGGAATGCTCAAGGCCTACC 3779
OQ632304_TuYV-ITA2      CGTTCGGGCCGAGTCTATCAGACTGCCCGGCATTCTCTAATGGAATGCTCAAGGCCTACC 3777
                        ************************************************************

OQ632303_TuYV-ITA1      ATGAGTATAAAATCTCAATGGTCATTTTGGAGTTCGTCTCCGAAGCCTCTTCCCAAAATT 3839
OQ632304_TuYV-ITA2      ATGAGTATAAAATCTCAATGGTCATTTTGGAGTTCATCTCAGAGGCCTCTTCCCAAAGTT 3837
                        *********************************** **** ** ************* **

OQ632303_TuYV-ITA1      CCGGTTCCATCGCTTACGAGCTGGACCCACACTGTAAACTCAACTCCCTTTCCTCAACTA 3899
OQ632304_TuYV-ITA2      CCGGTTCCATCGCTTACGAGCTGGACCCACACTGTAAACTCAACTCCCTTTCCTCAACAA 3897
                        ********************************************************** *

OQ632303_TuYV-ITA1      TCAACAAGTTCGGGATCACAAAGCCCGGGAAAAGGGCGTTTACAGCGTCTTACATCAATG 3959
OQ632304_TuYV-ITA2      TTAACAAATTCGGGATCACAAAGCCCGGGAAGAGGACGTTTACAGCGTCTTACATCAACG 3957
                        * ***** *********************** *** ********************** *

OQ632303_TuYV-ITA1      GAACGGAATGGCACGACGTTGCCGAGGACCAATTCAGGATCCTCTACAAAGGCAATGGTT 4019
OQ632304_TuYV-ITA2      GGGCGGAATGGCACGACATTGCCGAGGACCAATTCAGGATCCTCTACAAAGGCAATGGTT 4017
                        *  ************** ******************************************

OQ632303_TuYV-ITA1      CTTCATCGATAGCTGGTTCTTTCAGAATCACCATTAAGTGTCAATTCCACAACCCCAAAT 4079
OQ632304_TuYV-ITA2      CCTCATCGCCAGCCGGTTCTTTCAGGATCACCATAAGATGCCAATTCCACAACCCCAAAT 4077
                        * ******  *** *********** ******** *  ** *******************

OQ632303_TuYV-ITA1      AGGTAGACGAGGAACTCGGCCCTAGCCCAGGGCCTTCTCCCTCTCCACAACCCACACCCC 4139
OQ632304_TuYV-ITA2      AGGTAGACGAGGAACCTAGCCCTAGTCCAGGGCCAAGCCCCCAACCAACACCAACACCAA 4137
                        ***************   ******* ********    ***   ***  *** *****

OQ632303_TuYV-ITA1      AAAAGAAATATCGTTTTATCGTCTATACTGGAGTCCCCGTGACTCGTATAATGGCTCAAT 4199
OQ632304_TuYV-ITA2      CCAAAGAGTACAGATGGATAGTTTACACTGGCGTCTGTAGAACTCTAATAAAAGCCCAGA 4197
                          **  * **  * *  ** ** ** ***** ***      ****  ****  ** **

OQ632303_TuYV-ITA1      CTACGGATGATGCCATCTCTTTGTGTGATATGCCGTCCCAACGGTTCCGCTACATAGAGG 4259
OQ632304_TuYV-ITA2      CAACGGACGATTCAATCTATCTGTACGATCTGGGTTCCCAACGCCTCCGGTATATTGAAA 4257
                        * ***** *** * **** * ***  ***  **   ******** ****  ** ** **

OQ632303_TuYV-ITA1      ACGAAAACATGAACTGGACGAACCTCGATTCTCGATGGTATTCCCAGAATTCTTTGAAAG 4319
OQ632304_TuYV-ITA2      ATGAAAACATGAACTGGACCAACGTGGACGCCCGCTGGTATTCAAACAGCAATGTTAAAG 4317
```

```
                            * **************** *** * **   * ** ********  * *     * * ****

OQ632303_TuYV-ITA1          CCATCCCGATGATAATAGTGCCAGTCCCTCAAGGTGAGTGGACTGTGGAAATTTCGATGG 4379
OQ632304_TuYV-ITA2          CGGTTCCCATGTATGTGTTTACAGTCCCGGAAGGAACATGGTCAGTTGAGATATCAACAG 4377
                            *  * ** ***     *  *  *******  ****     *** * ** ** ** ** *  *

OQ632303_TuYV-ITA1          AGGGGTATCAACCAACCTCAAGCACCACAGATCCTAACAAGGACAAACAAGATGGTCTCA 4439
OQ632304_TuYV-ITA2          AAGGTTACCAACCTACAGCAAGTACCACTGACCCAAACAAGGGAAAGGTTGATGGCATGA 4437
                            * ** ** ***** **   **** ***** ** ** *******  **     *****  * *

OQ632303_TuYV-ITA1          TCGCTTACAACGATGATCTTAGTGAAGGTTGGAACGTGGGGATTTACAACAATGTGGAGA 4499
OQ632304_TuYV-ITA2          TTGCCTATTCTGATGATCAATCAGAAGTGTGGAATGTTGGAATAAATCAAAACTGTAACA 4497
                            * ** **    *******      ****  ***** ** ** **  *  * **     * *

OQ632303_TuYV-ITA1          TAACCAACAACAAGGCCGATAATACTTTGAAGTATGGCCACCCAGACATGGAACTCAATG 4559
OQ632304_TuYV-ITA2          TCACAAACCTGAAAGCAGACAATTCCTGGAAGTATGGGCACCCTGATATGGAAATAAACA 4557
                            * ** ***    ** ** ** *** * * ********* ***** ** ****** * **

OQ632303_TuYV-ITA1          GCTGTCATTTCAATCAAGGACAGTGTCTGGAAAGAGATGGAGATTTGACTTGTCATATCA 4619
OQ632304_TuYV-ITA2          ATTGCCACTTCAACCAGGGACAGGTATTGGAAATGGATGGTACAGTCTCCTTTCACGTTG 4617
                              ** ** ***** ** ******     ******  *****      *  * * ***  *

OQ632303_TuYV-ITA1          AGACGACTGGTGACAATGCCTCCTTCTTTGTTGTTGGACCCGCTGTCCAGAAGCAATCTA 4679
OQ632304_TuYV-ITA2          AGACCACTGGTGACAATGCATCATTTTTCCTAGTTGGTCCGGCAGTCCAAAAGTTGTCAA 4677
                            **** ************** ** ** **   * ***** ** ** ***** ***   ** *
                                                                    TuYV2-f1>
OQ632303_TuYV-ITA1          AGTATAATTACGCCGTTTCGTACGGAGCCTGGACAGATCGGATGATGGAGATAGGGATGA 4739
OQ632304_TuYV-ITA2          AATACAATTACGCCGTCTCATATGGAGCGTGGACAGACCGTGATATGGAGTTGGGCTTGA 4737
                            * ** *********** ** ** ***** ******** **     ****** * **  ***

OQ632303_TuYV-ITA1          TCGCCATAGCACTTGATGAACAAGGCTCATCCGGTTCCGCAAAGACAGAAAGACCAAAGA 4799
OQ632304_TuYV-ITA2          TCACAGTTTCTTTGGATGAAAGAGACGAGTCAAGAGGTTCCGCCCTGAAAAGACCTCGTC 4797
                            ** *  *  *  * ******  ** *   **  *     *        *******
                                                                 TuYV2-f2>
OQ632303_TuYV-ITA1          GAGTTGGGCACTCCATGGCAGTCTCAACCTGGGAGACTATAAAATTGCCGGAGAAGGAAA 4859
OQ632304_TuYV-ITA2          GAGAAGGACACTCGAAGGCAGTCTCCACCTGGGAGACTATAAACTTACCGGAGAAGGAAA 4857
                            ***   ** ***** * ********* **************** ** *************
                              TuYV1-f2>
OQ632303_TuYV-ITA1          ACTCCGAGGGATGCGAAACCAGTCAAAGACAAGACTCTAAAACTCCTCCCACAGCTAGTG 4919
OQ632304_TuYV-ITA2          ACTCCGAGAAAACTGAAACCGGTCAAAGACAAGACCTTAAAACTCCTGTTTTAATCAAAA 4917
                            ********  *   ****** **************  **********      *   *

OQ632303_TuYV-ITA1          GGGGTTCCGACACGCTGGACGTCGAAAAAGGAGGCTTGCCCCTTCCTGTTGAAGAAGAAA 4979
OQ632304_TuYV-ITA2          CAGTGTCTGATTCTGGTTCAGACGCAGGTTCTGCAC------------AGGACAATGAAA 4965
                              *  ** **  *       * ** *      *                 **  * ****
                                       TuYV2-f2*> TuYV2-f1*>
OQ632303_TuYV-ITA1          TCCCTGATTTTGTTGGGGATAACCCTTGGTCTGACTTATCGACTA------AGAATTCAC 5033
OQ632304_TuYV-ITA2          TCCCTGACAACTTGGAAGATGATCCTTGGGGAGGCATCACGGAGGCTAGCAAGCGTCCTG 5025
                            *******     * *  *** * ******   * * *  **           **   * *

OQ632303_TuYV-ITA1          AGGAAGAAGAGGCT---------------ATGTCGTCAAAGAGTGGTCTTAGACCCCAGT 5078
OQ632304_TuYV-ITA2          AAACAGAAGATGATGTCCCTCCTCAAGAGACCAAGTCTATCGGTGCTCTTGAACGCTTCA 5085
                            *   ****** * *               *    *** *   *** ****  ** *
                                          TuYV1-f1>
OQ632303_TuYV-ITA1          TGCAGCCTCCTGGTCTGCCAAAACCTCAACCAATCAGAACGATTCGAAACTTCGATCCAA 5138
OQ632304_TuYV-ITA2          AGCGGTCGGGCAAACCACCCTTAAGAGGCCCCAACCGAACAGTGACTACATTTCAACCCG 5145
                             ** * *       *  **   *      ** * * ****  *    *  **   * **

OQ632303_TuYV-ITA1          CACCGGATT---TGGTTGAAGCATGGCGACCCGATGTGAACCCCGGATATTCCAAAGCAG 5195
OQ632304_TuYV-ITA2          AGAGTGATCTCTTGGAGGCTTGGGATCCACCACATTTCAACCCCGGTTATACCAAAGAAG 5205
```

```
                             ***    ***  *        * ***  ** * ******** *** ****** **

OQ632303_TuYV-ITA1      ATGTGGCAGCTGCCACTGTCATCGTCGGGGGTTCCATCAAAGACGGCCGTTCTATGATTG 5255
OQ632304_TuYV-ITA2      ACGTCGCAGCAGCGACGATCTTAGCGGGTGGAGATGTTTGGGATGGCAGGGAGATGCTTC 5265
                        * ** ***** ** **  ** * *  ** **     *    ** *** *    *** **
                                          <TuYV2-r2*    <TuYV2-r1*  <TuYV2-r1

OQ632303_TuYV-ITA1      ATAAACGAAACAAAGCTGTGTTAGACGGTCGCAGGAGTTGGGGTTCCTCCTTGGCTTCC- 5314
OQ632304_TuYV-ITA2      AGAAACGAGATGATCAAATTCAAAGGAAGCGCAAATCCTGGGTTCCCTCTTTTGGAGGAA 5325
                        * ****** *  *     *   *      ****     **** * **** ** *
                                                      <TuYV2-r2           <TuYV1-r1
OQ632303_TuYV-ITA1      -----------TCCCTCACGGGTGGAACGCTCAAGGCCTCCGCAAAGTCGGAGAAGCTTG 5363
OQ632304_TuYV-ITA2      GTAAAACATCTACTCTTCAAGGTGGCTCTTTGCGACAAAGTAAAAACAGTGAGGCTTTAT 5385
                                    * **    *****  *  *            ***    ***    *

OQ632303_TuYV-ITA1      CCAAACTTACCACGAGTGAAAGGGCAAGGTATGAACAGATTAAGCGTCAGCAAGGCTCCA 5423
OQ632304_TuYV-ITA2      CTCACTTAACCACAGAGCAAAGACGCGAATATGAGGGAATTAAACGCCAGCAAGGTACGA 5445
                        *  *  * *****     ****       *****    ***** ** ********  * *
                                                                        <TuYV1-r2
OQ632303_TuYV-ITA1      CAAGAGCCTCAGAATTCCTAGAATCACTTCTAGCTGGCGAAGACCCCGACTCAAGGTTC- 5482
OQ632304_TuYV-ITA2      CACGAGCTTCAGAATTCTTAAAGCGGATTCTCGCTGGCAGTGAACCTTAACCTAAAACAG 5505
                        ** **** ********* ** *     **** ******   ** **  *  * *

OQ632303_TuYV-ITA1      ----T------------------------------GAAGGGATACAACTTGACCCTTCCC 5508
OQ632304_TuYV-ITA2      TTTCCACAGTACTCAACTGTGCTCGTTGGTAGTGATACGAGGCGAAAATCAAAACTACCC 5565
                                                            * * *    ** *  *  ** ***

OQ632303_TuYV-ITA1      GGTCCAGATGAACCTGTCCAAATCATCATCGTCAAGCCAGGGACTTTAAACTGGAACGAA 5568
OQ632304_TuYV-ITA2      ATTTTAGCATTAAATATTATAGCTAATAAAGTCAAGCCAGAGACATTAAACTGGAACGAC 5625
                          *  **    *  * *   *   *  *  ********** *** **************

OQ632303_TuYV-ITA1      TCCGTTTTACGGATAGGCAACGAGTGTTTTACGCTGGGAGAAATCCCTACGGCACTTCGG 5628
OQ632304_TuYV-ITA2      TCCGCTTTGCGGATAGGCAACGAGTGTTTT------------------------------ 5655
                        **** *** *********************

OQ632303_TuYV-ITA1      TGT       5631
OQ632304_TuYV-ITA2      ---       5655
```

Figure S3. Results of the BLAST analysis genomic region comprise between the nucleotides 4905 and 5503, showing the highest nucleotide diversity (π) in the TuYV-ITA2 isolate.

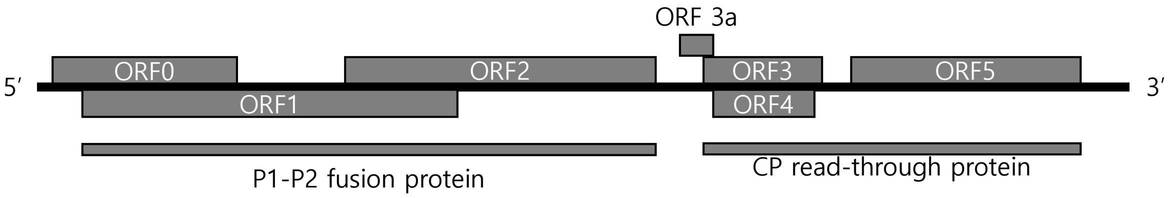

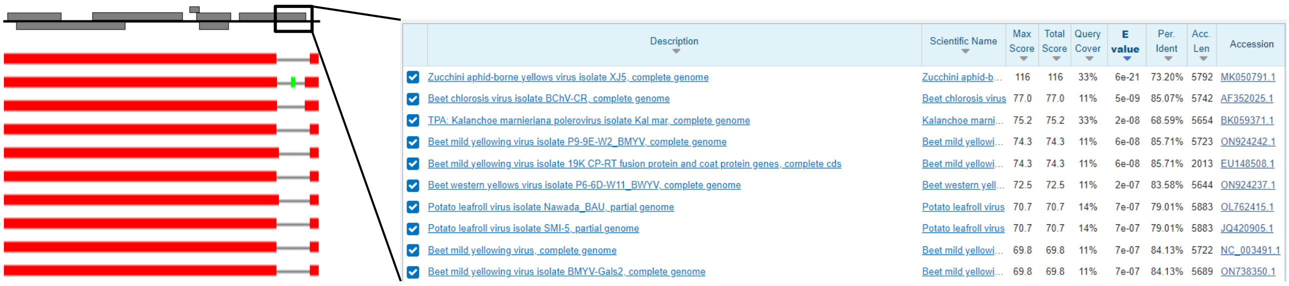

Description	Scientific Name	Max Score	Total Score	Query Cover	E value	Per. Ident	Acc. Len	Accession
Zucchini aphid-borne yellows virus isolate XJ5, complete genome	Zucchini aphid-b...	116	116	33%	6e-21	73.20%	5792	MK050791.1
Beet chlorosis virus isolate BChV-CR, complete genome	Beet chlorosis virus	77.0	77.0	11%	5e-09	85.07%	5742	AF352025.1
TPA: Kalanchoe marnieriana polerovirus isolate Kal mar, complete genome	Kalanchoe marni...	75.2	75.2	33%	2e-08	68.59%	5654	BK059371.1
Beet mild yellowing virus isolate P9-9E-W2_BMYV, complete genome	Beet mild yellowi...	74.3	74.3	11%	6e-08	85.71%	5723	ON924242.1
Beet mild yellowing virus isolate 19K CP-RT fusion protein and coat protein genes, complete cds	Beet mild yellowi...	74.3	74.3	11%	6e-08	85.71%	2013	EU148508.1
Beet western yellows virus isolate P6-6D-W11_BWYV, complete genome	Beet western yell...	72.5	72.5	11%	2e-07	83.58%	5644	ON924237.1
Potato leafroll virus isolate Nawada_BAU, partial genome	Potato leafroll virus	70.7	70.7	14%	7e-07	79.01%	5883	OL762415.1
Potato leafroll virus isolate SMI-5, partial genome	Potato leafroll virus	70.7	70.7	14%	7e-07	79.01%	5883	JQ420905.1
Beet mild yellowing virus, complete genome	Beet mild yellowi...	69.8	69.8	11%	7e-07	84.13%	5722	NC_003491.1
Beet mild yellowing virus isolate BMYV-Gals2, complete genome	Beet mild yellowi...	69.8	69.8	11%	7e-07	84.13%	5689	ON738350.1

Note to Figure 2 – Figure 2 was created by assembling three different agarose gels whose original photos are shown below:

Gel n. 1

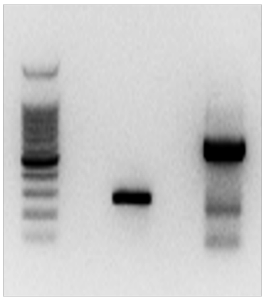

Gel n. 2

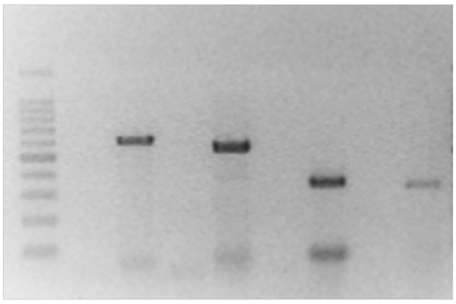

Gel n. 3

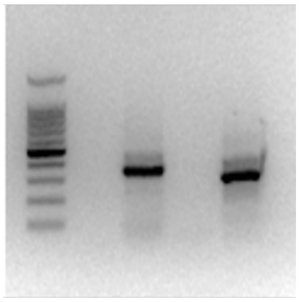

Supplement: Supplementary file 1 [file DataSheet1.pdf]
